# Analysis of genomes and transcriptomes of clear cell renal cell carcinomas identifies mutations and gene expression changes in the TGF-beta pathway

**DOI:** 10.3389/fgene.2022.953322

**Published:** 2022-09-15

**Authors:** Xiangyu Che, Jianyi Li, Yingkun Xu, Qifei Wang, Guangzhen Wu

**Affiliations:** ^1^ Department of Urology, The First Affiliated Hospital of Dalian Medical University, Dalian, Liaoning, China; ^2^ Organ Transplant Center, The First Affiliated Hospital, Sun Yat-sen University, Guangzhou, China; ^3^ Department of Endocrine and Breast Surgery, The First Affiliated Hospital of Chongqing Medical University, Chongqing, China

**Keywords:** TGF-β, ccRCC, TCGA, prognosis, treatment

## Abstract

The occurrence of clear cell renal cell carcinoma (ccRCC) is related to changes in the transforming growth factor-β (TGF-β) signaling pathway. In this study, we adopted an integrated approach to identify and verify the effects of changes in this pathway on ccRCC and provide a guide for identifying new therapeutic targets. We performed transcriptome analysis of 539 ccRCC cases from The Cancer Genome Atlas (TCGA) and divided the samples into different TGF-β clusters according to unsupervised hierarchical clustering. We found that 76 of the 85 TGF-β pathway genes were dysregulated, and 55 genes were either protective or risk factors affecting the prognosis of ccRCC. The survival time of patients with tumors with low TGF-β scores was shorter than that of patients with tumors with high TGF-β scores. The overall survival (OS) of patients with ccRCC with high TGF-β scores was better than that of patients with low TGF-β scores. The TGF-β score correlated with the expression of key ccRCC and deacetylation genes. The sensitivity of tumor patients to targeted drugs differed between the high and low TGF-β score groups. Therefore, a prognostic model based on the TGF-β gene pathway can predict the prognosis of ccRCC patients. Grouping patients with ccRCC according to their TGF-β score is of great significance for evaluating the prognosis of patients, selecting targeted drugs, and identifying new therapeutic targets.

## Introduction

As one of the most common cancers, the number of new cases and deaths of renal cell carcinoma (RCC) has remained high in recent years (Siegel et al., 2018; Siegel et al., 2019; Siegel et al., 2020). ccRCC accounts for 75%–80% of RCCs (Leibovich et al., 2010) and often warrants radiotherapy or chemotherapy (Coppin et al., 2011). At the time of preliminary diagnosis, 20%–30% of patients with RCC have local or distant metastases (Gong et al., 2016). Targeted drug therapy is a common treatment for these patients; however, one of the leading causes of treatment failure is drug resistance. Several studies have revealed the molecular mechanisms and signaling pathways of RCC, including TGF-β, Wnt-β-catenin, and angiogenesis signal transduction. The TGF-β signaling pathway, as one of the common signaling pathways, plays an important yet complex role in the occurrence and development of RCC and has a significant impact on tumor metastasis and prognosis (Kominsky et al., 2007; Wang et al., 2020a). At present, most studies suggest that the TGF-β pathway is involved in the regulation of tumors as a cancer-promoting factor (Bao et al., 2020; Du et al., 2020). However, some studies have shown that TGF-β can induce apoptosis in renal cancer cells, and c-Ski can weaken the anti-tumor effect of TGF-β by inhibiting TGF-β signal transduction (Taguchi et al., 2019).

Here, we used data from TCGA to systematically analyze the genetic changes, prognosis, and treatment-related information on TGF-β-related genes in RCC and to explore the role of the TGF-β signaling pathway in RCC.

## Materials and methods

### Data acquisition and analysis

Through the R/BioConductor package of TCGAbiolinks, we downloaded the RNA-seq transcriptome data on the ccRCC group from the Genomic Data Commons (GDC) portal (Colaprico et al., 2016); the data included 539 cases of ccRCC tissue and 72 cases of normal renal tissue. A total of 85 genes related to the TGF pathway were obtained from the Kyoto Encyclopedia of Genes and Genomes (KEGG) TGF-β signaling pathway on the Gene Set Enrichment Analysis (GSEA) website. The clinical information on the cancer patients was obtained from TCGAbiolinks, including the tumor size (T) status, metastatic (M) status, tumor grade, tumor stage, age, and survival status. The Lasso regression analysis was carried out with “glmnet” and “Survival” packages. Univariate and multivariate COX risk analyses of clinical features were performed with the “Survival” package. The correlation of immune infiltration was analyzed with the “ggstatsplot” package.

### Genetic alteration and survival analysis

The differential expression of TGF-β pathway genes in ccRCC and normal renal tissues was analyzed using the “limma” package, and the effect of TGF-β pathway genes on the prognosis of patients with RCC was analyzed using the “Survival” package. We downloaded the single-nucleotide variation (SNV) data and expression data on TGF-β pathway genes in different cancer types from TCGA database (Tomczak et al., 2015), analyzed them using Perl language, and visualized them with TBtools software (Chen et al., 2020).

### Cluster analysis based on transforming growth factor-β scores

We constructed a TGF-β scoring model to show the differences between samples. Based on the expression characteristics of normal renal tissues, we divided the renal carcinoma tissues into three categories: the TGF-β normal-score group (cluster 1), TGF-β high-score group (cluster 2), and TGF-β low-score group (cluster 3 and cluster 4). We used violin plots to describe the relationship between normal tissues and gene expression levels in these three groups. We plotted survival curves for the three clusters using the “Survival” package. We used “pheatmap” to draw a heat map showing the relationship between these three clusters and the clinicopathological features of ccRCC patients.

### Prediction of targeted drug response

We predicted the therapeutic response based on the Genomics of Drug Sensitivity in Cancer (GDSC) database (Yang et al., 2012). The R package “pRRophetic” was used for the prediction process; ridge regression was used to estimate the half-maximal inhibitory concentration (IC50) of the sample, and the 10-fold cross-validation based on the GDSC training set was used to evaluate the prediction accuracy (Geeleher et al., 2014a; Lu et al., 2019). All parameters were set to default values with the removal of the batch effect of “combat” and tissue type of “allSoldTumours,” and duplicate gene expression was summarized as the mean value (Geeleher et al., 2014b). The multiple testing correction used was a Bonferroni adjustment.

### Immune cell infiltration and immunotherapy

We analyzed immune cells quantitatively using the single-sample gene set enrichment analysis (ssGSEA) combined with the expression of TGF-β-related genes (Zhang et al., 2018). The heat map was drawn using “ggplot2” and “dplyr” in R. We used five R software packages, “ggplot2,” “dplyr,” “data.table,” “tidyr,” and “ggstatsplot” to analyze and plot the correlation between the TGF-β score and immune factors. We selected two classical immune regulators: type II interferon response and mast cells. Then, we used the “ggdisterstats” package to show their correlation with the TGF-β score in the form of a scatter plot. Then, we adopted the “ggdisterstats” package to make scatter diagrams to show both of their correlations with the TGF-β score. Visual correlation matrix analysis was used to show the relationship between programmed cell death 1(PD1 and PDCD1), cytotoxic T-lymphocyte-associated protein-4 (CTLA-4), and TGF-β scores. We used the subclass mapping and tumor immune dysfunction and rejection (TIDE) algorithm to predict the clinical response of RCC to block immune checkpoints PD-1 and CTLA-4 (Hoshida et al., 2007; Jiang et al., 2018). Bonferroni correction was used in this process.

### Establishment of the Lasso regression prognostic model

Based on its statistical significance, we first selected the TGF-β gene related to survival (*p* < 0.05). We then used a Lasso regression analysis to delete genes that may overfit the model. Finally, a multivariate analysis was used to determine the optimal predictive factors of the model. The analysis used the following formula: Risk score = Σ Ni = 1 (Expi × Coei). The median was set as the cut-off value, according to which all patients with ccRCC were divided into two groups: the low-risk group and high-risk group. The overall survival time-dependent recipient operating characteristics were used to evaluate the accuracy of the prognostic model. Taking the median as the cut-off value, all patients with ccRCC were divided into the low-risk group and high-risk group. The overall survival time-dependent recipient operating characteristics were used to evaluate the accuracy of the prognostic model. To evaluate the accuracy of the prognostic model, we adopted the OS time-dependent receiver operating characteristic (ROC) curve.

### Compounds targeting transforming growth factor-β pathway genes

The Baird Institute’s public online Connectivity Map (CMAP) Build02 (Lamb et al., 2006) (https://portals.broadinstitute.org/cmap/) can predict compounds that activate or inhibit targets based on gene expression signatures, and we used this tool to explore which drugs may target TGF-β pathway genes. We further used the CmapTools to conduct a special analysis to explore the mechanism of action of the compound (https://clue.io/) (Subramanian et al., 2017). Similar to the GSEA analysis, CMAP uses the pattern-matching strategy of the Kolmogorov–Smirnov test to find the similarity between differentially expressed genes (DEGs). Then, we compared the results of the CMAP analysis to the DEG ranking list to determine the positive or negative regulatory relationship of genes and to generate enrichment scores (ES) from −1 to 1. Finally, we used the aforementioned scores to rank all the case data in the database. After we obtained two tables in each type of tumor, we applied the results of the connection map to the expression signatures of the TGF-β pathway and then used *p* < 0.05 as our inclusion criteria to determine the average meaningful compounds for each type of tumor.

## Results

### Transforming growth factor-β pathway genes were significantly differentially expressed in clear cell renal cell carcinoma samples compared with normal samples and were closely related to prognosis

**FIGURE 1:**
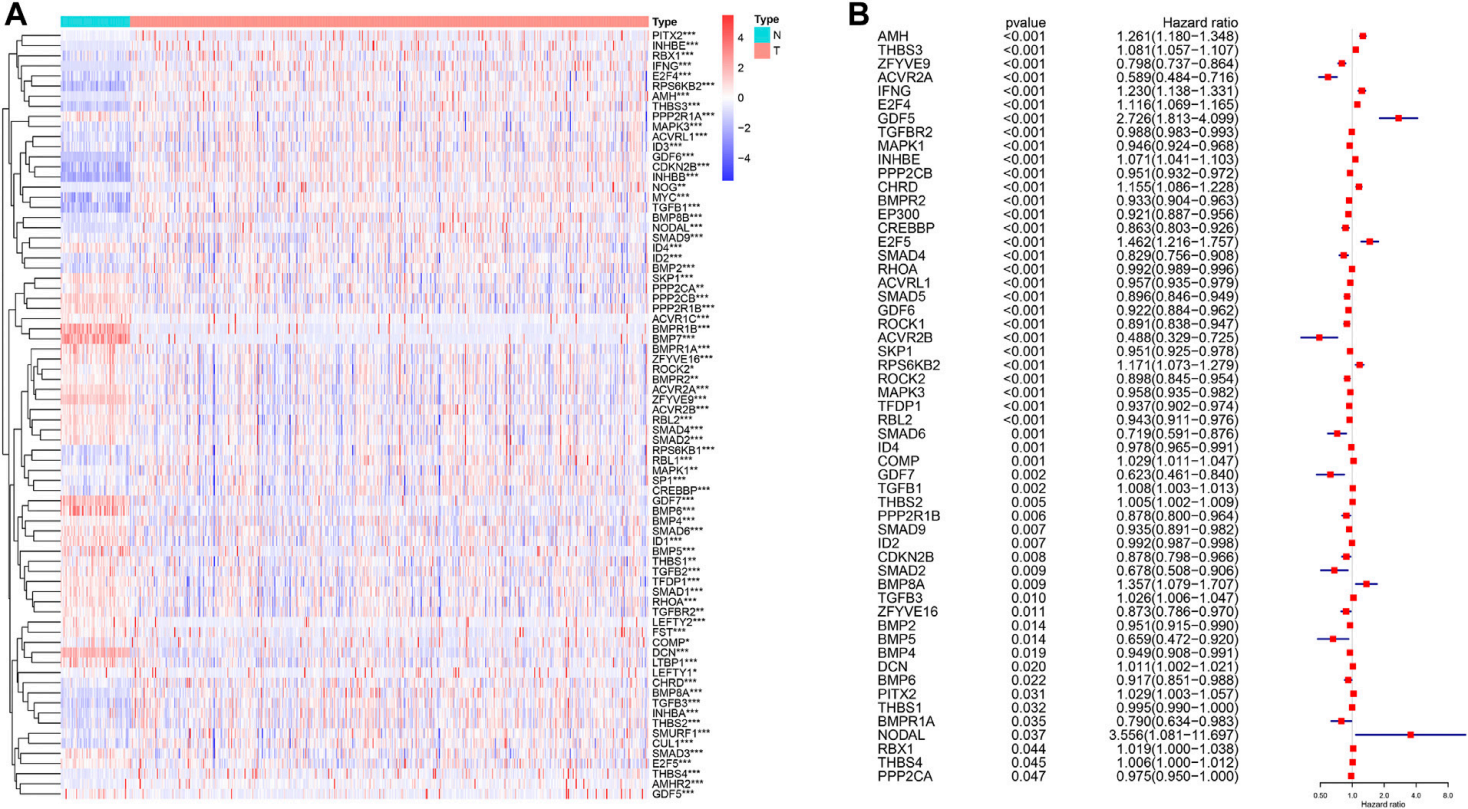
Differential expression of TGF-β pathway genes in ccRCC and their effect on prognosis. **(A)** Dysregulation of the TGF-β pathway genes in ccRCC. N: normal kidney tissue, T: kidney tumor tissue. Blue represents a low level of gene expression, red represents a high level of gene expression, and the color depth represents the level of gene expression. *p < 0.05, **p < 0.01, and ***p < 0.001. **(B)** TGF-β pathway genes that affect the prognosis of patients with ccRCC. A hazard ratio <1 represents a protective factor for prognosis, while a hazard ratio >1 represents a risk factor for prognosis. ccRCC: clear cell renal cell carcinoma.

From the KEGG TGF-β signaling pathway on the GSEA website, we found 85 genes related to the TGF-β signaling pathway (Supplementary Table S1) and analyzed the RNA sequencing data on 539 patients with human ccRCC and 72 normal kidney samples. We found that 76 genes of the TGF-β pathway were dysregulated in ccRCC compared with normal kidney tissues, of which 39 genes were upregulated and 37 were downregulated (Figure 1A; Supplementary Table S2). A total of 36 genes were upregulated and 40 were downregulated compared with those in normal kidney tissues. Furthermore, we analyzed the effects of these DEGs related to the TGF-β pathway on the overall survival of patients with ccRCC. The results showed that 55 genes significantly affected the prognosis of patients with ccRCC, of which 19 genes were risk factors (hazard ratio >1) and 36 genes were protective factors (hazard ratio <1) (Figure 1B; Supplementary Table S3).

### Unsupervised hierarchical clustering and prognostic analysis

**FIGURE 2:**
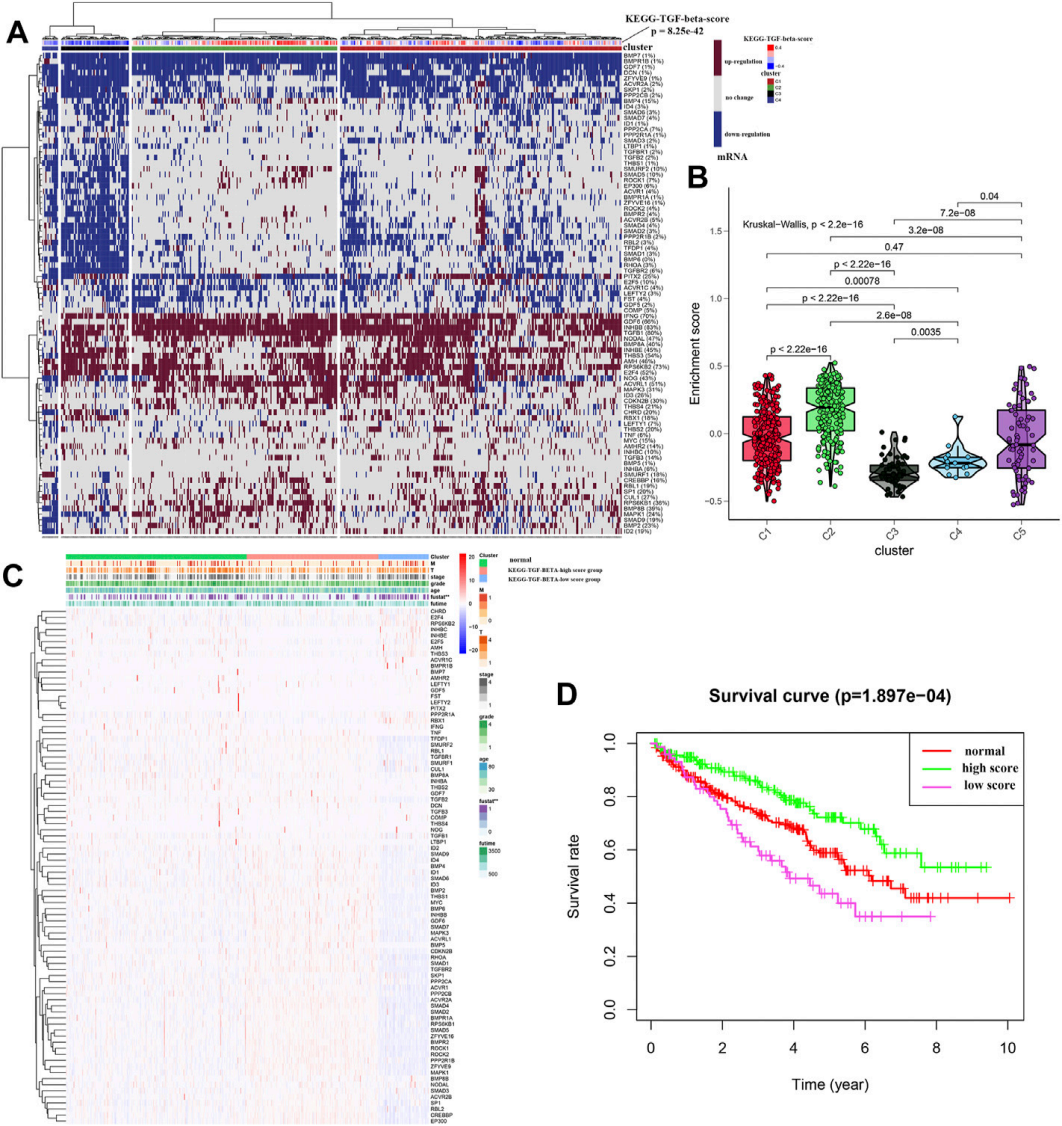
Unsupervised hierarchical clustering of ccRCC samples. **(A)** Cluster analysis of transcriptome data from 539 ccRCC samples from TCGA. **(B)** Box plot showing the activity score of the TGF-β pathway in each of the four clusters. **(C)** Relationship between the risk score and clinicopathological features. **(D)** Kaplan–Meier survival curves of the high-, low-, and normal-score clusters. *p < 0.05, **p < 0.01, and ***p < 0.001.

Unsupervised hierarchical clustering of the data revealed four different clusters of the TGF-β pathway (Figure 2A). Cluster 1 showed that the expression levels of 85 genes were similar to those in the normal samples, indicating that the TGF-β pathway was in a normal state. However, the expression levels of genes related to the TGF-β pathway in cluster 2 were increased, indicating high TGF-β scores, while cluster 3 and cluster 4 showed low expression of TGF-β pathway genes, indicating low TGF-β scores. Figure 2B depicts the TGF-β scores in five clusters (where cluster 5 represents normal kidney tissue) and further shows the differences in TGF-β scores between them. Supplementary Table S4 shows the TGF-β scores for each sample. We then further classified all of the samples into three groups; the normal group, the KEGG-TGF-β high-score group, and the KEGG-TGF-β low-score group. In other words, clusters 3 and 4 were merged into the latter group. We then analyzed the correlation between the TGF-β score and clinicopathological features, and the results showed that the expression of TGF-β pathway genes was significantly correlated with the overall survival rate of patients with ccRCC (Figure 2C). Further survival analysis showed that the KEGG-TGF-β low-score group had the worst prognosis, while the high-score group had the best prognosis (Figure 2D).

### Disruption of the transforming growth factor-β signaling pathway is closely related to dysregulation of key and deacetylated genes in clear cell renal cell carcinoma

**FIGURE 3:**
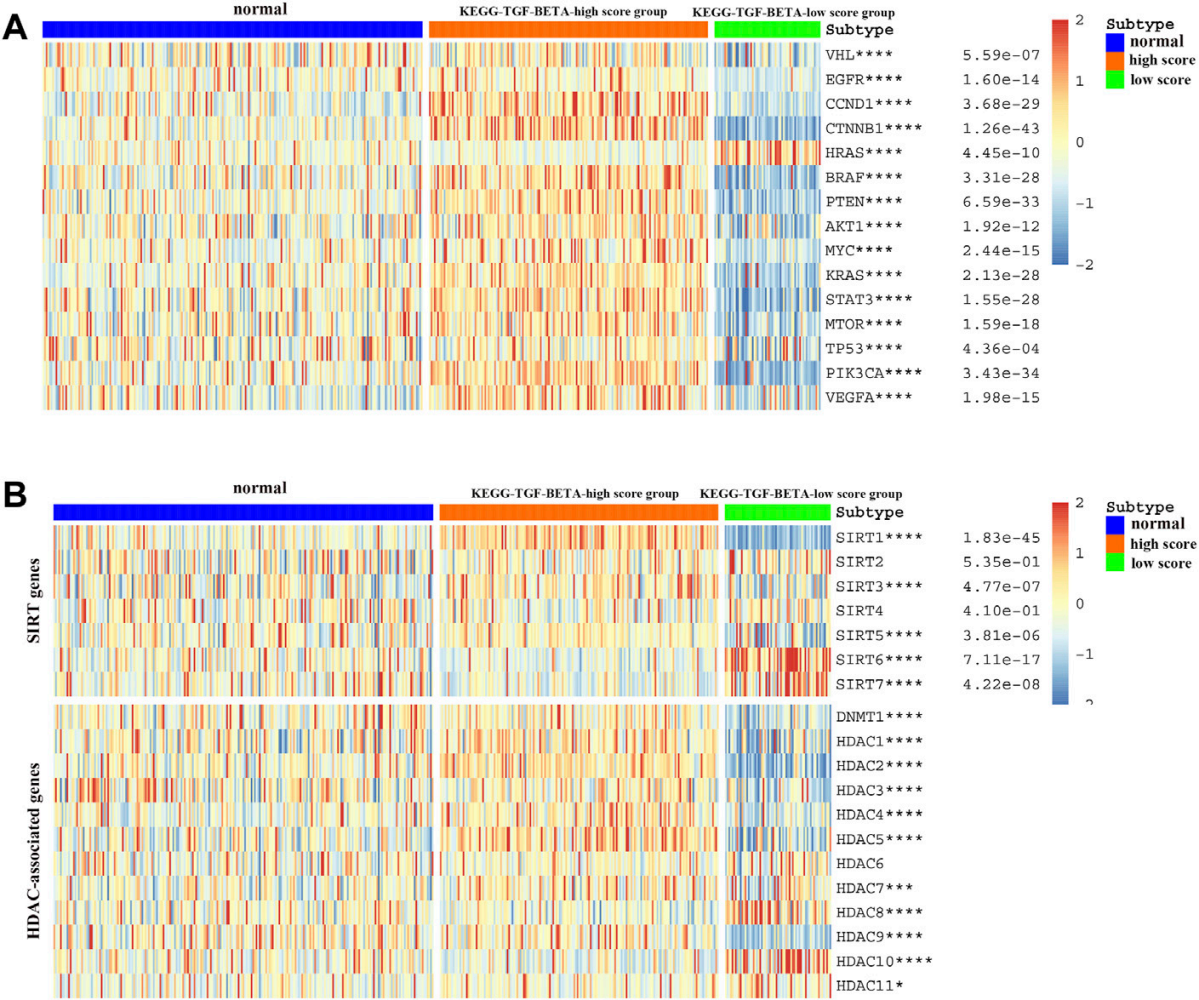
Relationship between the TGF-β score and the expression of other key genes. **(A)** Relationship between the activation level of the TGF-β pathway and the expression of oncogenes and tumor suppressor genes. **(B)** Relationship between the activation level of the TGF-β pathway and the expression of deacetylation genes. Blue represents the TGF-β normal-score group, orange represents the TGF-β high-score group, and green represents the TGF-β low-score group. *p < 0.05, **p < 0.01, ***p < 0.001, and ****p < 0.0001.

We explored the relationship between the expression of various well-known key genes and the TGF-β pathway in ccRCC (Figure 3A). In other words, VHL, TP53, and PTEN were downregulated in the KEGG-TGF-β low-score group. These results suggest that disruption of the TGF-β signaling pathway is related to the promotion of tumors. *EGFR*, *MYC*, *VEGFA*, and other oncogenes were highly expressed in the KEGG-TGF-β high-score group, indicating that it may be more effective to target these genes in this group. Analysis of the transcriptome of ccRCC patients also showed a strong correlation between the abnormal expression of sirtuins and histone deacetylases (HDACs) and the abnormality of the TGF-β pathway (Figure 3B).

### Prediction of IC50 of different targeted drugs based on the transforming growth factor-β score

**FIGURE 4:**
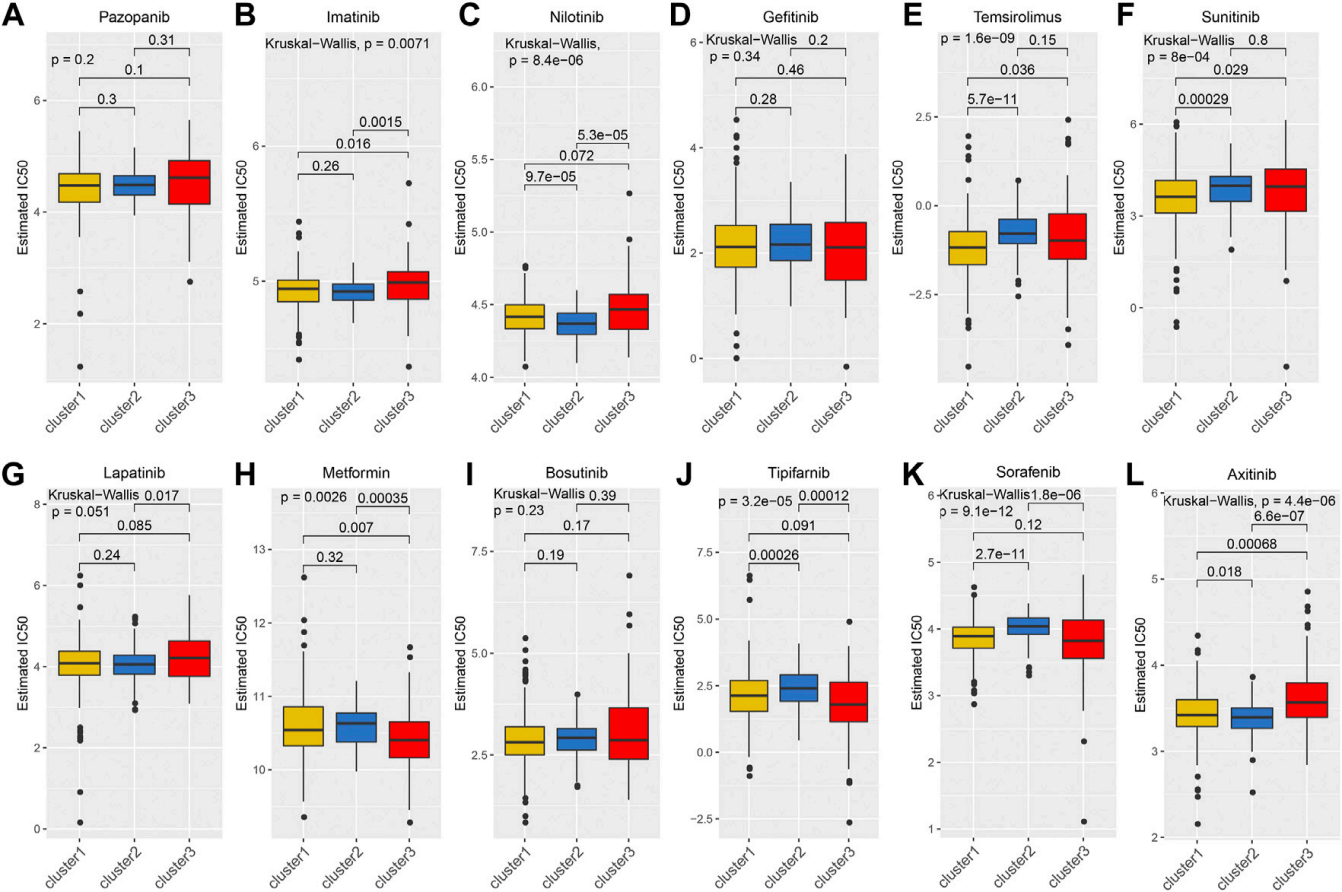
Prediction of IC50 values of targeted drugs. **(A)** pazopanib, **(B)** imatinib, **(C)** nilotinib, **(D)** gefitinib, **(E)** temsirolimus, **(F)** sunitinib, **(G)** lapatinib, **(H)** metformin, **(I)** bosutinib, **(J)** tipifarnib, **(K)** sorafenib, and **(L)** axitinib.

Considering that targeted drugs are commonly used in the treatment of metastatic RCC, we evaluated the efficacy of different targeted drugs on the TGF-β signaling pathway in the KEGG-TGF-β high-score and low-score groups. We obtained a satisfactory prediction model using ridge regression on the GDSC cell-line dataset. Based on this model, we evaluated the IC50 values of 12 targeted drugs. The results of the analysis showed that there was no significant difference in the IC50 values of pazopanib, gefitinib, bosutinib, and lapatinib among the three groups (Figures 4A,D,G,I). Compared with the KEGG-TGF-β high-score and low-score groups, IC50 of temsirolimus and sunitinib was lower in the normal group (Figures 4E,F). Therefore, it is recommended that these two drugs be used for the treatment of the normal group. The IC50 values of imatinib, nilotinib, and axitinib were lower in the KEGG-TGF-β high-score group than those in the normal and the KEGG-TGF-β low-score groups (Figures 4B,C,L). This indicates that these three drugs may have better results in the KEGG-TGF-β high-score group. Compared with the normal and KEGG-TGF-β high-score groups, the IC50 values of metformin, tipifarnib, and sorafenib were lower than those in the KEGG-TGF-β low-score group (Figures 4H,J,K). This indicates that three drugs may have better results in the KEGG-TGF-β low-score group.

### The transforming growth factor-β pathway is related to immune regulation

**FIGURE 5:**
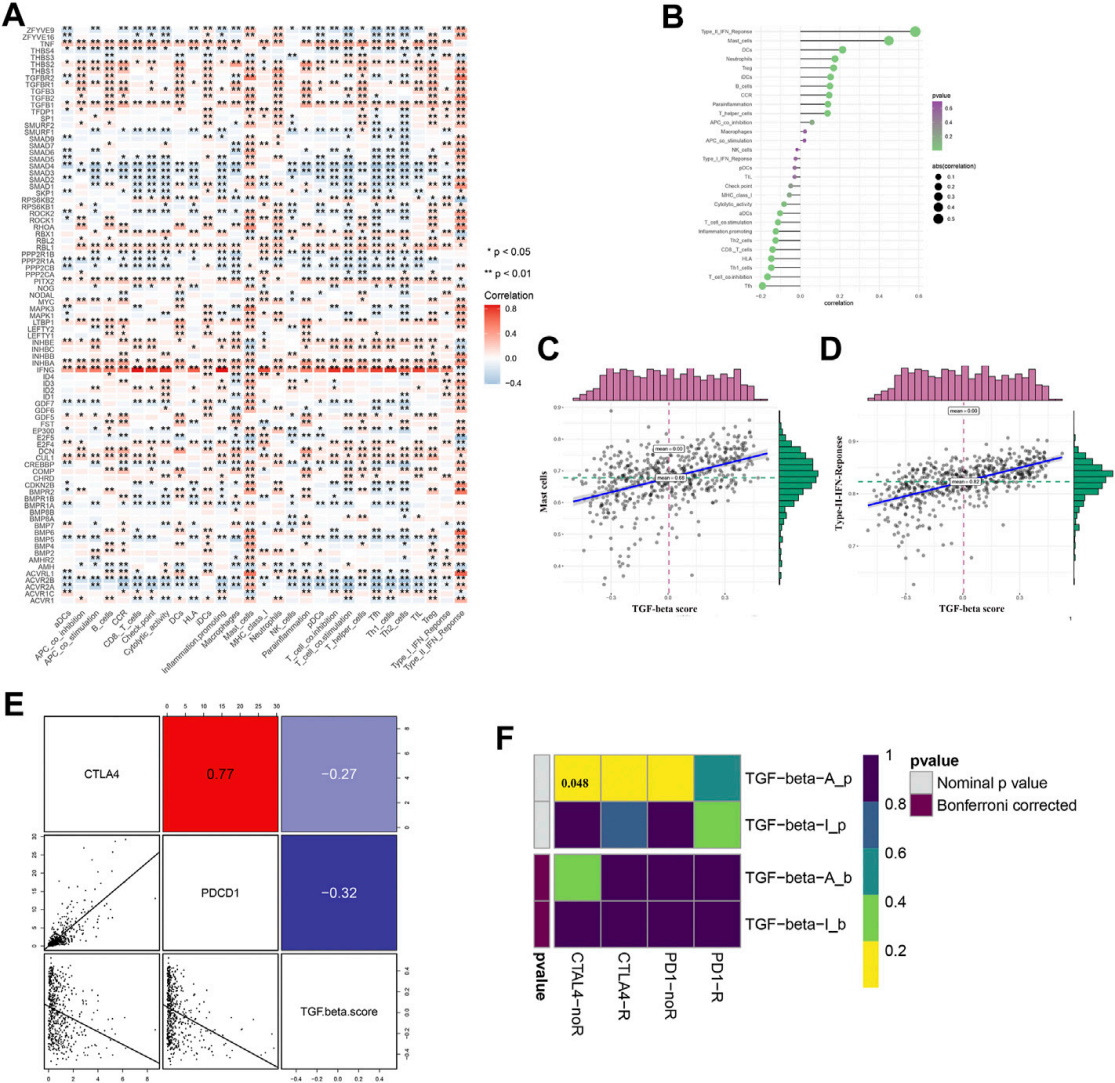
Relationship between TGF-β pathway genes and immune regulation. **(A)** Relationship between TGF-β pathway genes and immune cell infiltration. **(B–D)** Relationship between the TGF-β score and immune infiltration. **(E)** Relationship between the TGF-β score and immune checkpoint expression (Pearson’s correlation analysis). **(F)** Prediction of immune checkpoint inhibitor response in TGF-β activation and inactivation groups. Blue represents a positive correlation, and red represents a negative correlation. Pearson’s correlation analysis was used. *p < 0.05 and **p < 0.01.

Immune regulation plays a vital role in the tumor microenvironment. We identified that many TGF-β genes were associated with the infiltration of many types of immune cells (Figure 5A). We then analyzed the correlation between the immune infiltration and TGF-β score and found that there was a close relationship between them (Figure 5B), especially Type-II-IFN-response and mast cells, which were positively correlated with the TGF-β score (Figures 5C,D). Next, we analyzed the correlation between the TGF-β score and immune checkpoints, and the results showed that the former was negatively correlated with CTLA4 and PDCD1 (Figure 5E). We used the TIDE algorithm to predict the response of the KEGG-TGF-β low-score and high-score groups to immune checkpoint inhibitors; however, after Bonferroni correction, we did not find a significant difference in the response of the groups to immune checkpoint inhibitors (Figure 5F).

### Construction and verification of a new transforming growth factor-β-based survival model

**FIGURE 6:**
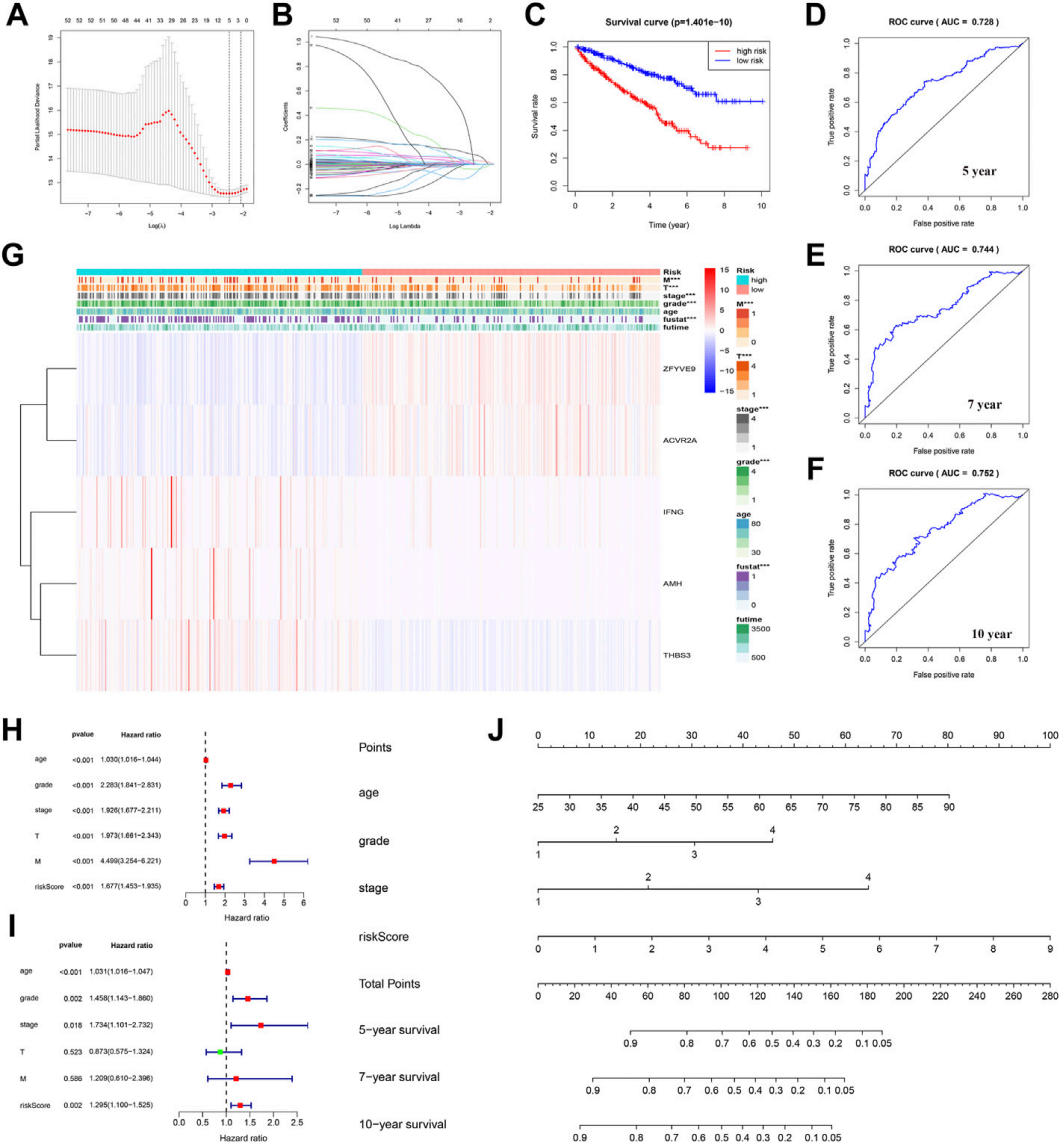
Establishment of a prognostic model based on TGF-β in ccRCC. **(A)** Partial likelihood of deviance was plotted against log(lambda). **(B)** Lasso coefficient profiles of TGF-β in ccRCC. (**C**) Grouped according to the risk scores calculated by the new survival model based on TGF-β expression; the Kaplan–Meier survival curve shows the overall survival rate of ccRCC patients in both the high- and low-risk groups. **(D–F)** ROC curve analysis showed that the new survival model was efficient in predicting prognosis, and the AUC values for 5-, 7-, and 10-year survival were 0.728, 0.744, and 0.752, respectively. **(G)** Relationship between the risk score and clinicopathological features. **(H)** Univariate Cox regression analysis showed that clinicopathological parameters such as age, grade, stage, tumor size (T), tumor metastasis (M), and the risk score of the new survival model were correlated with OS in patients with renal cell carcinoma (RCC). **(I)** Multivariate Cox regression analysis showed that risk score, age, grade, and stage were independent risk factors affecting the prognosis of patients with ccRCC. **(J)** New nomogram predicted 5-, 7-, or 10-year survival rates in patients with ccRCC. The value of each variable is a fraction along the dotted axis. The nomogram has nine lines. The second, third, fourth, and fifth lines represent the age, grade, stage, and risk score, respectively. The total score in the sixth row is derived from the sum of each score of age, grade, stage, and risk score. Based on the total score of ccRCC patients, the 5-, 7-, and 10-year survival rates of patients can be estimated.

First, TGF-β genes related to survival were screened according to the survival analysis and significance value (*p* < 0.05). The Lasso regression model was then used to analyze and determine the most reliable prognostic markers. The number of points at which the vertical line intersects the curve at the same site in Figure 6A is the number of variables for when the fit is optimal, which indicates the number of selected genes. On this basis, five genes *ZFYVE9*, *ACVR2A*, *IFNG*, *AMH*, and *THBS3* were selected to establish a risk-characteristic model based on the minimum criterion (Figures 6A,B). Then, according to the median risk score, we divided ccRCC patients into low- and high-risk groups and studied the prognostic performance of the new survival model composed of five genetic risk characteristics. Kaplan–Meier survival analysis revealed that patients in high-risk groups had a significantly lower survival rate than patients in low-risk groups (Figure 6C). In addition, ROC curve analysis was performed to analyze the prognostic performance of the new survival model in patients with ccRCC. The area under the curve (AUC) value was 0.728 for the 5-year survival rate, 0.744 for the 7-year survival rate, and 0.752 for the 10-year survival rate. Therefore, the prognostic model of ccRCC based on the TGF-β pathway has a relatively high predictive value (Figures 6D–F). To better understand the relationship between clinicopathological characteristics of ccRCC patients and TGF-β pathway genes, we systematically analyzed the correlation between risk scores based on five TGF-β pathway genes and clinicopathological characteristics of ccRCC patients. We found that the risk score was closely related to (T) and (M) status, tumor grade, stage, and OS (Figure 6G). Univariate Cox regression analysis showed that these features along with the risk score were associated with overall survival in ccRCC patients (Figure 6H; Supplementary Table S5). Multivariate Cox regression analysis showed that age, grade, stage, and risk score were independent risk factors affecting the prognosis of patients with ccRCC (Figure 6I; Supplementary Table S6). We used a nomogram to predict the ccRCC risk; 5-year, 7-year, and 10-year survival rates were estimated based on the patient’s age, grade, stage, and risk score (Figure 6J).

### Transforming growth factor-β pathway genes undergo a wide range of genetic changes across cancer types and affect the prognosis of many cancers

**FIGURE 7:**
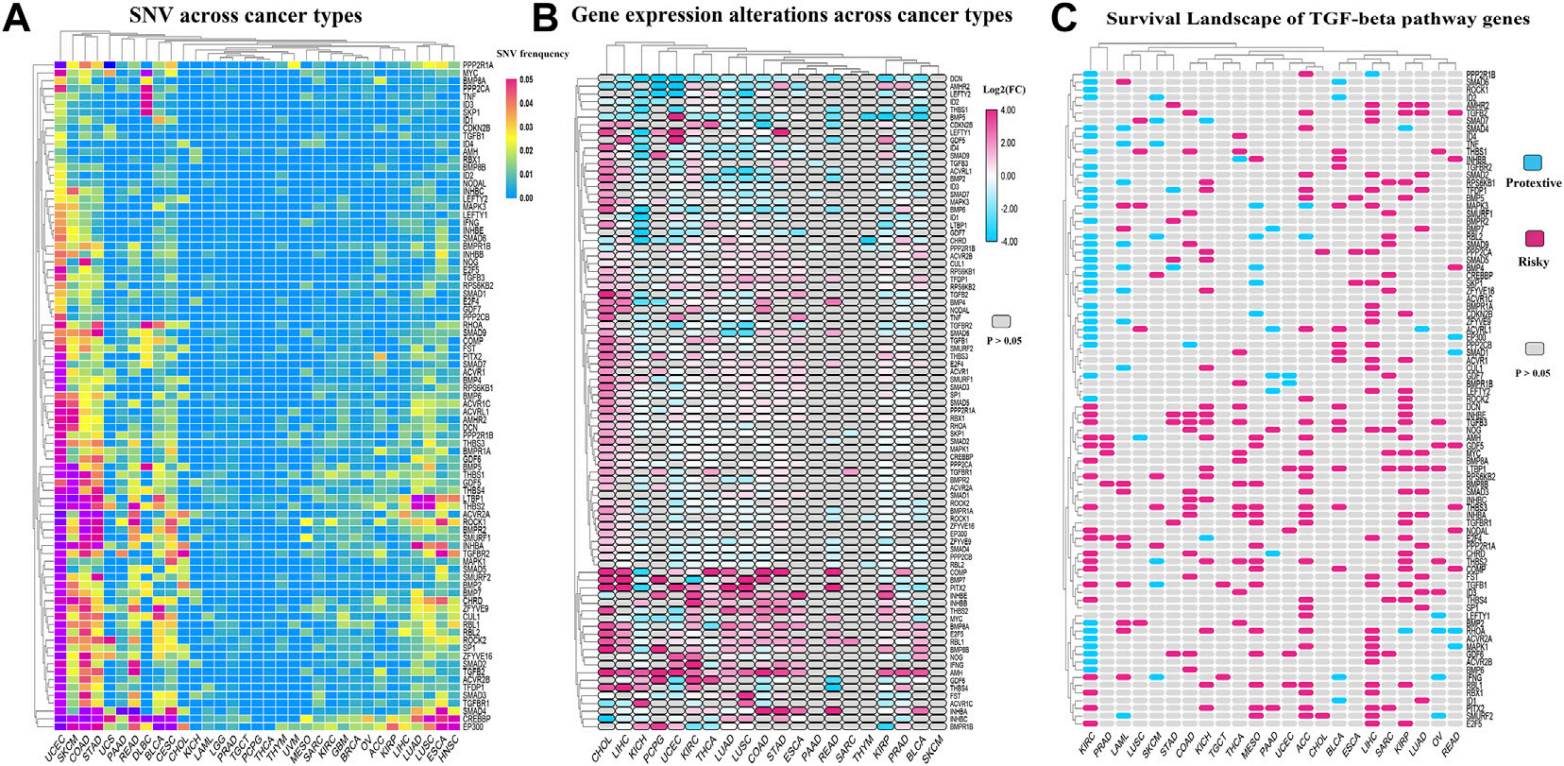
Genetic changes of TGF-β pathway genes across cancer types. **(A)** SNVs of TGF-β pathway genes across cancer types. **(B)** Alterations in the expression of TGF-β pathway genes across cancer types. **(C)** Risk assessment of the effect of the TGF-β pathway genes on prognosis. KIRC: kidney renal clear cell carcinoma

To further explore the genetic changes in TGF-β pathway genes in pan-tumors, we analyzed SNV and gene expression changes of TGF-β pathway genes across multiple cancer types. The results showed that TGF-β pathway genes had a wide range of SNVs (Figure 7A; Supplementary Table S7) and gene expression differences (Figure 7B; Supplementary Table S8) across the different cancer types. We then analyzed the effect of TGF-β pathway genes on the prognosis of cancer patients (Figure 7C; Supplementary Table S9). The results showed that most of the TGF-β pathway genes were risk factors in other types of tumors, but were protective factors in ccRCC, which was consistent with our previous analysis that the KEGG-TGF-β low-score group had a worse prognosis.

### Connectivity map analysis identified potential compounds/inhibitors targeting TGF-β

**FIGURE 8:**
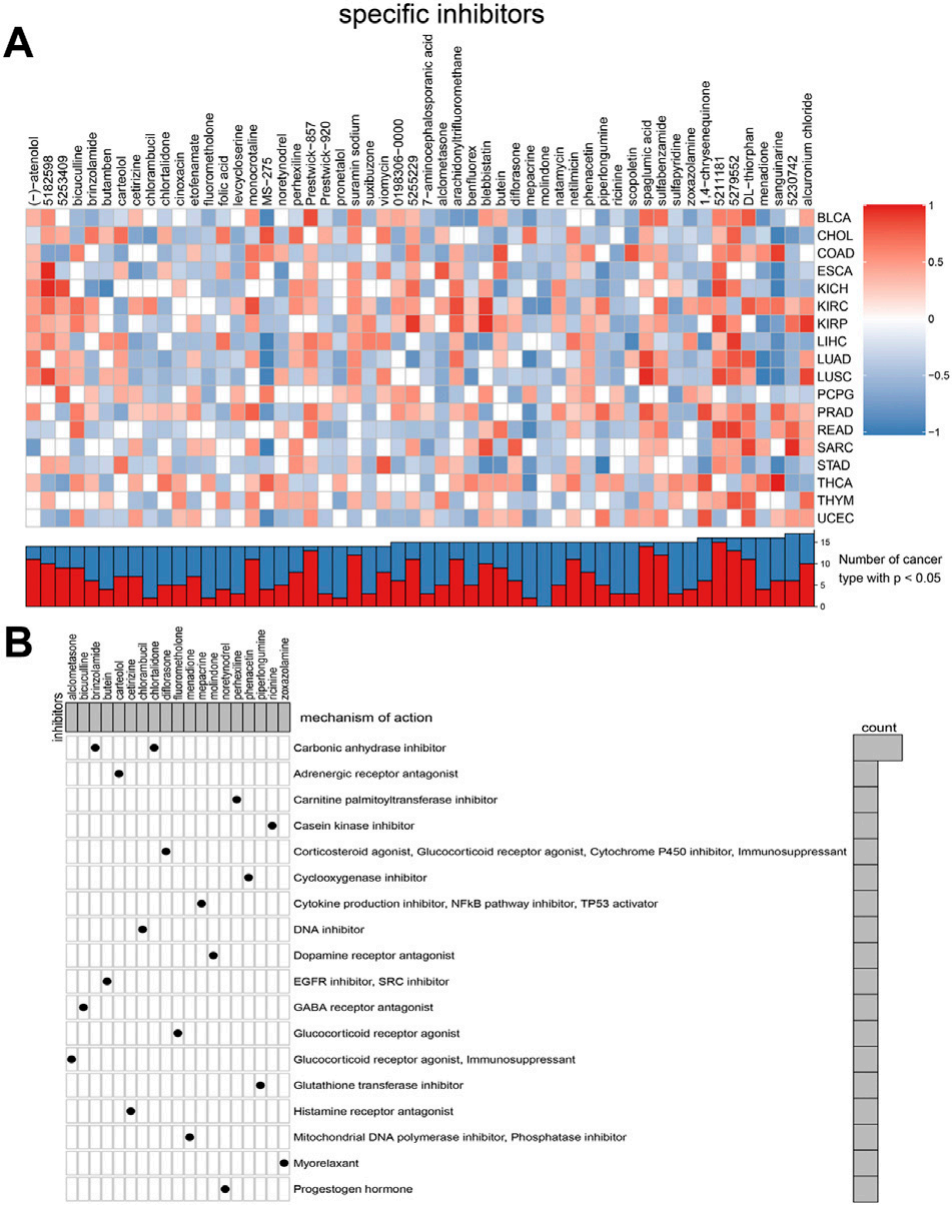
Connectivity map (CMAP) analysis identified potential compounds/inhibitors targeting TGF-β. **(A)** Potential inhibitors of TGF-β were predicted using CMAP. In the upper part of Figure 8A, the ordinate represents the type of tumor and the abscissa represents the name of the compound. Red indicates that the compound inhibits the TGF-β signaling pathway, whereas blue indicates that the compound promotes the TGF-β signaling pathway. The color depth represents the intensity of inhibition or promotion. In the lower half of Figure 8A, the red part represents the number of cancer types in which the compound inhibited the TGF-β signaling pathway. **(B)** Exploration of the mechanism of small molecular compounds. KIRC: kidney renal clear cell carcinoma.

Considering that most TGF-β pathway genes are risk factors in tumors, we aimed to identify compounds that can inhibit TGF-β pathway genes. We used a data-driven systematic method, CMAP, to explore the relationship between genes, compounds, and biological conditions to identify candidate compounds that may target TGF-β pathway genes. We found 54 compounds inhibiting TGF-β pathway genes that were enriched in different tumors (Figure 8A). Simultaneously, we explored the possible action mechanisms of 19 small molecular compounds and found that the compounds involved 18 mechanisms, where two compounds had the same mechanism (Figure 8B). Therefore, this suggests that we can select different compounds in different tumors and suppress the TGF-β pathway genes, according to different mechanisms to achieve tumor inhibition.

## Discussion

Our comprehensive analysis of a large number of open access RCC cases provides new insights into the key role of the TGF-β pathway in the occurrence and development of RCC. Previous studies have reported the effects of various TGF-β pathway genes on RCC. For example, TGF-β1 enhances the proliferation and metastatic potential of RCC cells by upregulating lymphoid enhancer-binding factor 1/integrin αMβ2 (Liu and Shang, 2020), and MUC12 relies on TGF-β1 signaling to mediate the growth and invasion of renal cancer cells (Gao et al., 2020).

However, we found that when we analyzed all genes of the TGF-β pathway as a whole, the results were surprising. Analysis of TCGA database revealed that 76 of the 85 TGF-β pathway genes were significantly differentially expressed between RCC and normal renal tissues, and 55 genes could play a pivotal role in the prognosis of patients with RCC. The results of this analysis piqued our interest in the role of TGF-β pathway genes in RCC.

In the three groups that we divided the ccRCC samples into (normal, KEGG-TGF-β high-score, and KEGG-TGF-β low-score), the degree of TGF-β gene expression, prognosis, and response to drugs differed. Similar to hepatocellular carcinoma (Chen et al., 2018), the prognosis of the TGF-β high-score group was better and that of the low-score was poorer in RCC.

We observed a correlation between the expression of many well-known genes related to RCC and the expression of TGF-β pathway genes. The VHL gene was expressed at significantly lower levels in the KEGG-TGF-β low-score group, and the loss of VHL gene function often leads to the pathogenesis of RCC. Similarly, the expression levels of well-known tumor suppressor genes such as PTEN and P53 were also low in the KEGG-TGF-β low-score group, which explains the poor prognosis of this group. However, a related study showed that the synergistic effect of TGF-β type I receptor and hypoxia-inducible factor-α (HIF-α) promotes the progression of RCC (Mallikarjuna et al., 2019). There are some obvious differences between this study and our research results, which may warrant further study. In the KEGG-TGF-β low-score group, we found some highly expressed oncogenes, such as *EGFR*, *MYC*, *MTOR*, and *VEGFA*, which play a key role in the occurrence and development of RCC. Therefore, compared with the KEGG-TGF-β low-score group, patients in the KEGG-TGF-β high-score group may have a better therapeutic outcome if these oncogenes are used as therapeutic targets.

Acetylation and deacetylation are common epigenetic modifications that play vital roles in the formation and development of tumors. Analysis of TCGA database transcriptome also showed a strong correlation between the aberrant expression of sirtuins and HDACs and the abnormal expression of the TGF-β pathway in patients with RCC. SIRT1, SIRT3, and SIRT5 were significantly correlated with a high TGF-β score, while the expression levels of SIRT6 and SIRT7 were significantly correlated with a low TGF-β score. In the KEGG-TGF-β low-score group, the expression of SIRT3 was significantly low. Previous studies have shown that low SIRT3 expression in RCC is associated with poor prognosis (Jeh et al., 2017), which supports our results. Transcriptome analysis of sirtuins and HDACs also indicated that the expression of these proteins in different TGF-β feature groups was different; therefore, selecting different targets in different TGF-β feature groups may be more effective for therapy.

Currently, targeted drug therapy is commonly used for local or distant metastatic RCC, and it is still worth discussing which targeted drug can benefit patients the most. The vasculature-rich nature of RCC has led to the approval of tyrosine kinase inhibitors, including sorafenib, sunitinib, pazopanib, and axitinib (Escudier et al., 2007; Motzer et al., 2007; Rini et al., 2011; Motzer et al., 2014), targeting the VEGF signaling axis as first- and second-line therapies for metastatic RCC in the United States and the European Union. Pazopanib as a first-line targeted agent is similar to sunitinib in improving progression-free survival (PFS) and overall survival (OS) (Motzer et al., 2013; Motzer et al., 2014). The choice between these two agents for patient treatment is a matter of debate, as with other agents. We attempted to address this issue using TGF-β scores in patients with renal cancer. We classified the samples according to KEGG-TGF-β scores and predicted the IC50 values of various targeted drugs. We observed that the sensitivities of the three groups to the targeted drugs were different. This suggests that choosing different targeted drugs according to the different patient characteristics can afford better efficacy or appropriately reduce the drug concentration to lessen the side effects of the drug. This provides a guide for a more detailed classification of patients according to their different characteristics, which will result in patients receiving more personalized treatment and ultimately improve the effectiveness of treatment.

Immunotherapy is a popular topic in the field of tumor therapy. TGF-β has systemic immunosuppressive effects and inhibits host immunosurveillance (Yang et al., 2010). Exploring the relationship between TGF-β and immunity will help us gain more insight into TGF-β-targeted therapy or immunotherapy. Our results show that the expression of TGF-β pathway genes is closely related to immune cell infiltration, and type-II-IFN-response and mast cells were most related to TGF-β scores. This discovery may be of great significance for the development of new or improved immunotherapy regimens. Two immune checkpoint inhibitors, PD-1 and CTLA-4, are the main drugs approved by the Food and Drug Administration for the treatment of advanced RCC (Xu et al., 2017; Rini et al., 2019). Recent studies have found that the combination therapy of blocking TGF-β and PD-1/PD-L1 has achieved relatively ideal efficacy (Lind et al., 2019; Wang et al., 2020b). It has also been found that selective inhibition of TGF-β1 produced by GARP-expressing Tregs can overcome resistance to PD-1/PD-L1 blocking in cancer (de Streel et al., 2020).

The TGF-β score was negatively correlated with the expression of CTLA4 and PDCD1, which indicated that the TGF-β low-score group had higher expression of these proteins, and blocking CTLA4 and PDCD1 immune checkpoints may have a better therapeutic effect in this group. However, there was no significant difference in the response of the TGF-β high-score and low-score groups to anti-CTLA4 and anti-PDCD1 therapy after Bonferroni correction. Further research is needed on the classification of TGF-β gene expression in RCC to guide immunity and on the close relation of TGF-β expression to immune cell infiltration and the expression of PD-1 and CTLA4.

IFN-γ, the only type-II interferon, is a key cytokine produced by activated T cells and natural killer (NK) and NK-T cells in the tumor microenvironment. IFN-γ signals play an important role in coordinating processes (Ayers et al., 2017) such as anti-cancer immunity, improving tumor immunogenicity, and causing anti-tumor effects through the host immune system (Castro et al., 2018). The main function of PD-1 is to weaken the response of effector T cells and prevent the escape of tumor cells from immune attack (Chen et al., 2019). However, IFN-γ signaling can ultimately induce feedback inhibition, compromising anti-tumor immunity. However, IFN-γ has another role. As part of the feedback loop, IFN-γ signals activate the PD-1 signal axis (Ayers et al., 2017). The main reason why the TGF-β score is positively correlated with IFN and negatively correlated with PD-1 may be that the main effect of IFN is an anti-tumor effect, which may also imply the importance of maintaining a relative balance of components in the tumor microenvironment. Some studies have shown that in metastatic RCC, the OS and PFS rates of patients with high mast cell density are significantly better than those of patients with low mast cell density (Yao et al., 2021). This may explain the positive correlation between mast cells and the TGF-β score, but the role of mast cells in tumors is diverse (Cherdantseva et al., 2017; Yao et al., 2021), and the specific mechanism requires further study.

We used Lasso regression to establish a prognostic model of RCC based on the TGF-β pathway genes. The results showed that the prognosis in the low-risk group was significantly better than that in the high-risk group. Multivariate Cox regression analysis showed that the TGF-β score was an independent risk factor for RCC. These results once again prove the importance of the TGF-β pathway in the prognosis of RCC.

TGF-β pathway genes also have a wide range of SNV and gene expression alterations across multiple cancer types and play a role in a variety of tumor types as prognostic molecules. Consistent with our study, a previous study performed an integration analysis of TGF-β superfamily genetic alterations in 9,125 tumor samples from 33 cancer types, elucidated the salient characteristics of TGF-β-related genes in a large group of different cancer types, and found high-frequency genetic alterations in the TGF-β superfamily across cancer types (Korkut et al., 2018). TGF-β pathway genes are risk factors in most tumors, but in ccRCC, most TGF-β pathway genes are protective factors, which makes the role of TGF-β pathway genes in KIRC different from that in other tumors. Therefore, the different characteristics of TGF-β pathway genes in RCC warrant further investigation.

This study has some limitations. Although the TGF-β score is closely related to immune cell infiltration and immune checkpoint expression, we found that the response of the TGF-β-activated and -inactivated clusters to immune checkpoint inhibitors was not statistically significant, which means that the TGF-β score cannot be used to guide immunotherapy in ccRCC patients. Moreover, our gene set did not include some downstream TGF-β signaling target genes, such as the EMT-related genes *CDH1*, *CDH2*, *SNAI1*, and *VIM*, which makes our investigation of pathway activity imperfect. Because we classified the samples mainly based on the TGF-β score, these genes were not within the TGF-β pathway given by KEGG, so we could not give TGF-β scores for these genes. As such, these genes were excluded.

In summary, compared with normal renal tissues, most genes of the TGF-β pathway are significantly differentially expressed in ccRCC and can serve as risk or protective factors that affect the prognosis of patients with ccRCC. People with low TGF-β scores have a worse prognosis, and most genes of the TGF-β pathway are involved in the regulation of ccRCC as protective factors. Stratifying patients with RCC according to their TGF-β score is of great significance for evaluating the prognosis of patients and finding new targets.

## Data Availability

All the code used to generate the analysis results is available at the following address https://github.com/ljy11652/ljy11652.git.
