# Supplementary material for: Analysis of genomes and transcriptomes of clear cell renal cell carcinomas identifies mutations and gene expression changes in the TGF-beta pathway
Source: Front Genet. 2022 Sep 15;13:953322. doi: 10.3389/fgene.2022.953322 (PMC9519989; doi:10.3389/fgene.2022.953322)
Supplement: Supplementary file 1 [file DataSheet1.PDF]

Table s1

KEGG_TGF_BETA_SIGNALING_PATHWAY
> TGF-beta signaling pathway
AC023512.1
ACVR1
ACVR1C
ACVR2A
ACVR2B
ACVRL1
AMH
AMHR2
BMP2
BMP4
BMP5
BMP6
BMP7
BMP8A
BMP8B
BMPR1A
BMPR1B
BMPR2
CDKN2B
CHRD
COMP
CREBBP
CUL1
DCN
E2F4
E2F5
EP300
FST
GDF5
GDF6
GDF7
ID1
ID2
ID3
ID4
IFNG
INHBA
INHBB
INHBC
INHBE
LEFTY1
LEFTY2
LTBP1
MAPK1

MAPK3
MYC
NODAL
NOG
PITX2
PPP2CA
PPP2CB
PPP2R1A
PPP2R1B
RBL1
RBL2
RBX1
RHOA
ROCK1
ROCK2
RPS6KB1
RPS6KB2
SKP1
SMAD1
SMAD2
SMAD3
SMAD4
SMAD5
SMAD6
SMAD7
SMAD9
SMURF1
SMURF2
SP1
TFDP1
TGFB1
TGFB2
TGFB3
TGFBR1
TGFBR2
THBS1
THBS2
THBS3
THBS4
TNF
ZFYVE16
ZFYVE9

Table s2

TGF-beta pathway genes expression alterations in ccRCC

gene	conMean	treatMean	logFC	pValue
TFDP1	16.05437823	13.67813242	-0.23109553	5.03E-07
SMURF2	4.917843649	5.04106879	0.035703773	0.97589999
SMURF1	6.21142798	7.578410816	0.286970373	1.95E-06
ROCK1	6.58990319	6.675990261	0.018724579	0.999716427
RPS6KB1	3.602003337	4.583004563	0.347494204	1.12E-15
PPP2R1A	45.36293041	39.97681327	-0.182350367	2.88E-06
ID2	46.24549791	66.60505313	0.526318711	1.52E-11
AMH	0.060116315	0.39390046	2.712002619	5.55E-17
INHBC	0.115921714	0.182724957	0.65652286	0.710603506
SMAD6	2.264816865	1.720753458	-0.396353988	6.68E-08
THBS1	75.45209908	59.25656509	-0.348586037	0.007328517
DCN	37.83822894	6.456298357	-2.551065409	3.60E-34
BMP8B	0.62984977	1.481873557	1.234342685	3.49E-09
CHRD	1.449497498	2.320202885	0.678698121	0.000162922
SMAD5	9.086796872	8.790240529	-0.047869186	0.091582115
CUL1	15.12241259	18.42501916	0.284977798	1.46E-12
COMP	1.47414883	1.975074029	0.422024542	0.022316189
ACVR1	11.68009526	11.07245619	-0.077076751	0.119294227
SMAD3	10.52017964	9.068474845	-0.214227499	3.90E-05
MAPK3	19.01007667	23.90756592	0.330702903	1.73E-11
GDF7	2.2486763	0.819640688	-1.456012489	1.48E-32
SP1	16.37865182	19.5121696	0.252557594	6.05E-07
RHOA	187.2085394	161.6620071	-0.21166558	6.49E-07
RBL2	16.47055377	12.71542138	-0.37330979	1.12E-12
ACVR2A	3.213246412	2.373549331	-0.436985586	4.95E-18
LEFTY2	0.291210193	0.244021165	-0.255054571	3.57E-17
ZFYVE9	8.966857917	5.240273244	-0.774960498	1.54E-30
BMP8A	0.213770844	0.581148875	1.44284269	3.12E-15
IFNG	0.033548737	0.770130499	4.520772586	6.90E-30
RBL1	1.326733278	1.782458156	0.425989844	1.05E-09
ID1	58.31612812	34.04441485	-0.776476799	3.18E-10
E2F4	10.8192231	14.44577446	0.417050645	6.40E-19
SMAD7	13.21166319	12.78407519	-0.047464298	0.218941349
THBS3	2.824244795	6.733152931	1.253417092	3.20E-27
TGFBR1	15.75211511	14.20083637	-0.149569659	0.308903138
E2F5	1.573906595	1.388319369	-0.181010442	1.70E-07
MYC	12.39717526	35.5352638	1.519239974	3.09E-23
SKP1	26.69519011	21.23782546	-0.329943767	7.53E-15
ACVRL1	8.608416881	15.37851214	0.83709608	1.20E-14
PPP2CA	26.84690729	25.45588218	-0.076756839	0.005017201
TGFB1	14.84169031	43.80108281	1.561311126	8.28E-32
BMP5	0.843819919	0.34911068	-1.273250651	9.95E-07
SMAD4	6.799994698	5.819659393	-0.224598903	1.41E-08
RPS6KB2	3.135730155	5.378582638	0.778424631	6.08E-34
BMP6	14.46727746	3.283360439	-2.139548413	2.46E-19
PPP2CB	33.75077067	24.3345538	-0.471914124	1.39E-20
SMAD9	3.333165211	5.099934576	0.613585912	0.000675204
AMHR2	0.009813668	0.018848328	0.941572085	1.23E-06

SMAD1	3.669466568	2.803179931	-0.388506002	1.86E-09
ROCK2	7.871780342	7.191263242	-0.130444742	0.014750892
TGFB3	2.46405981	4.899647406	0.991640657	6.29E-15
BMPR2	14.72962124	13.18294185	-0.160047981	0.002558568
CDKN2B	1.077422873	3.489958433	1.695625256	1.72E-29
CREBBP	6.200256355	7.218105123	0.219292288	0.00030553
TNF	0.444833077	0.607347486	0.449258104	0.238728969
PPP2R1B	7.009968659	5.417747723	-0.371714778	2.36E-14
LEFTY1	0.866979279	1.487078766	0.778411646	0.010596315
ACVR1C	0.167109821	0.100788928	-0.729459366	1.07E-20
LTBP1	21.20703778	10.85219809	-0.966555831	2.60E-07
INHBB	1.785995587	25.17630702	3.817266256	5.40E-38
RBX1	12.97182347	16.03171126	0.305547135	3.52E-06
NODAL	0.042646223	0.132608872	1.636687424	3.60E-18
ID3	38.71560933	59.19492111	0.61255805	2.12E-07
GDF5	0.159991159	0.09592973	-0.737942278	4.56E-11
TGFBR2	76.1767154	64.89081269	-0.231335849	0.001012082
ACVR2B	1.207428603	0.987506955	-0.290075068	2.01E-08
ID4	23.55193341	17.80327547	-0.403702802	3.28E-06
PITX2	0.127979089	0.486394786	1.926219658	5.66E-06
BMPR1B	6.925853302	1.525887365	-2.182343358	1.89E-36
BMP4	4.337040044	4.31285783	-0.008066604	0.000703833
BMP7	3.334839872	0.378357416	-3.139795863	1.94E-37
EP300	12.74561625	11.97119126	-0.090434405	0.051673272
INHBE	0.114432546	1.186352417	3.373963305	7.23E-18
GDF6	0.378908269	4.544392681	3.584166973	2.78E-29
INHBA	1.92317074	3.56746346	0.891411801	6.02E-08
BMP2	3.691079952	6.790559167	0.87948739	7.52E-11
BMPR1A	3.162446804	2.354014885	-0.42591777	3.30E-14
THBS4	0.536691158	2.644459094	2.300808633	0.000172947
ZFYVE16	5.932706905	4.629614245	-0.357798522	3.79E-09
THBS2	8.586893882	22.59929676	1.396069612	7.09E-07
NOG	0.237738854	1.549986069	2.704805639	0.003555884
TGFB2	2.187457729	1.654942696	-0.402473876	8.14E-05
FST	1.380861168	1.583529909	0.197575838	1.16E-10
SMAD2	2.638506934	2.268058785	-0.218263742	2.42E-09
MAPK1	18.47070253	20.73861247	0.167080633	0.009174601

Table s3

Univariate Cox regression analysis of the risk of TGF-beta pathway genes in ccRCC.

id	HR	HR.95L	HR.95H	pvalue
AMH	1.261406276	1.180413686	1.347956069	6.95E-12
THBS3	1.08139587	1.056808287	1.106555505	2.58E-11
ZFYVE9	0.798132439	0.737414493	0.863849838	2.33E-08
ACVR2A	0.589093025	0.484419542	0.716384378	1.15E-07
IFNG	1.230444733	1.137737444	1.330706173	2.12E-07
E2F4	1.115786373	1.068766174	1.164875218	6.12E-07
GDF5	2.725758883	1.812733636	4.098650427	1.45E-06
TGFBR2	0.987881036	0.982921735	0.992865359	2.05E-06
MAPK1	0.94583866	0.924331589	0.96784615	2.09E-06
INHBE	1.071205986	1.040763346	1.102539081	2.92E-06
PPP2CB	0.951395653	0.9315491	0.971665035	3.61E-06
CHRD	1.15478309	1.085565698	1.228413892	5.04E-06
BMPR2	0.933052015	0.904225249	0.962797781	1.51E-05
EP300	0.920798583	0.886717355	0.956189732	1.80E-05
CREBBP	0.862542549	0.80335478	0.926091023	4.56E-05
E2F5	1.461760769	1.216274567	1.756794562	5.18E-05
SMAD4	0.828777497	0.756055076	0.908494847	6.12E-05
RHOA	0.992423064	0.988632729	0.996227931	9.79E-05
ACVRL1	0.956662554	0.934951057	0.978878237	0.000155201
SMAD5	0.895953389	0.845937429	0.948926536	0.000177771
GDF6	0.922040279	0.883572878	0.962182405	0.000189197
ROCK1	0.890748699	0.838032894	0.94678055	0.000201623
ACVR2B	0.48844931	0.32924748	0.724630385	0.000370154
SKP1	0.951319697	0.925365561	0.978001781	0.000406135
RPS6KB2	1.171223001	1.072623802	1.278885772	0.000427529
ROCK2	0.897878367	0.845159897	0.953885253	0.000484426
MAPK3	0.958106663	0.93471044	0.982088504	0.000691713
TFDP1	0.937094856	0.902026898	0.973526146	0.000841573
RBL2	0.943129455	0.911188173	0.976190424	0.000866014
SMAD6	0.719268065	0.590741594	0.875757783	0.001035014
ID4	0.977966698	0.964817266	0.991295344	0.001256208
COMP	1.029194574	1.011224862	1.047483611	0.00136469
GDF7	0.622543741	0.461144929	0.840431469	0.001966055
TGFB1	1.008139988	1.002917773	1.013389395	0.002217139
THBS2	1.00526858	1.001575944	1.00897483	0.005131847
PPP2R1B	0.878015578	0.799848853	0.963821291	0.00624659
SMAD9	0.935013883	0.890728068	0.981501529	0.006644147
ID2	0.992443569	0.986977688	0.997939719	0.00710476
CDKN2B	0.878061464	0.797903601	0.966272033	0.007757897
SMAD2	0.677954248	0.507528965	0.905607351	0.00850934
BMP8A	1.357494088	1.07943024	1.707187858	0.008960689
TGFB3	1.026284933	1.006145271	1.046827724	0.010292894
ZFYVE16	0.873310636	0.786365511	0.969868917	0.011349224
BMP2	0.951405871	0.914507656	0.989792842	0.013574427
BMP5	0.658714946	0.471620916	0.920029974	0.014329315
BMP4	0.94904266	0.908473192	0.991423829	0.018957244
DCN	1.011216334	1.001748375	1.020773778	0.020129735
BMP6	0.916989206	0.851430053	0.987596339	0.022035989

PITX2	1.029300124	1.002661512	1.056646469	0.030878008
THBS1	0.994804887	0.99007232	0.999560076	0.032288736
BMPR1A	0.789507663	0.634139155	0.982942539	0.034528988
NODAL	3.556202233	1.08114808	11.69735632	0.036761888
RBX1	1.018892142	1.000497532	1.037624946	0.044064612
THBS4	1.006219627	1.000142672	1.012333506	0.044843215
PPP2CA	0.974668575	0.950288507	0.999674125	0.04712428

Table s4

Cluster		TGF-BETA-SCORE_score P = 8.25e-42
TCGA-A3-3306-01A-01R-0864-07	C1	-0.245542281
TCGA-A3-3329-01A-01R-0864-07	C2	0.130387001
TCGA-B8-5550-01A-01R-1541-07	C1	0.104826575
TCGA-A3-3378-01A-02R-1325-07	C1	-0.303358317
TCGA-A3-3343-01A-01R-0864-07	C1	-0.240219058
TCGA-B0-5100-01A-01R-1420-07	C1	0.346647001
TCGA-CJ-4869-01A-02R-1426-07	C1	-0.050381837
TCGA-CJ-4895-01A-01R-1305-07	C1	0.237608215
TCGA-B0-5691-01A-11R-1541-07	C2	0.335135229
TCGA-B0-5121-01A-02R-1420-07	C1	-0.009469832
TCGA-B0-5098-01A-01R-1420-07	C1	-0.207302736
TCGA-A3-3308-01A-02R-1325-07	C1	0.118118962
TCGA-BP-4807-01A-01R-1305-07	C2	0.353826302
TCGA-B0-5707-01A-11R-1541-07	C1	-0.111796043
TCGA-BP-4971-01A-01R-1334-07	C1	0.113147167
TCGA-B8-4620-01A-02R-1325-07	C1	0.067903986
TCGA-BP-4967-01A-01R-1334-07	C1	-0.005434644
TCGA-BP-5198-01A-01R-1426-07	C1	0.275528428
TCGA-BP-4326-01A-01R-1289-07	C1	0.159186365
TCGA-CJ-4904-01A-02R-1426-07	C2	0.411839644
TCGA-CZ-4853-01A-01R-1426-07	C1	-0.201356521
TCGA-A3-3387-01A-01R-1541-07	C1	0.219603566
TCGA-A3-3326-01A-01R-0864-07	C1	-0.14095003
TCGA-BP-4784-01A-01R-1305-07	C2	0.376408882
TCGA-BP-4766-01A-01R-1289-07	C2	-0.141529998
TCGA-CZ-5456-01A-01R-1503-07	C1	0.160085357
TCGA-CZ-4857-01A-01R-1305-07	C1	-0.006827649
TCGA-B0-5400-01A-01R-1503-07	C3	-0.212518802
TCGA-BP-5010-01A-02R-1420-07	C3	-0.033415789
TCGA-CJ-4644-01A-02R-1325-07	C2	0.172227461
TCGA-CJ-6033-01A-11R-1672-07	C1	0.086076497
TCGA-EU-5907-01A-11R-1672-07	C1	-0.017452478
TCGA-B4-5836-01A-11R-1672-07	C2	0.162791866
TCGA-A3-3323-01A-02R-1325-07	C1	-0.169981563
TCGA-BP-4353-01A-02R-1289-07	C1	0.12433447
TCGA-B0-5077-01A-01R-1334-07	C1	-0.085047676
TCGA-CZ-5984-01A-11R-1672-07	C1	-0.271900652
TCGA-B8-A54G-01A-11R-A266-07	C1	-0.40699052
TCGA-B0-4713-01A-01R-1277-07	C3	-0.304247118
TCGA-CZ-5463-01A-01R-1503-07	C2	0.039009158
TCGA-6D-AA2E-01A-11R-A37O-07	C1	-0.326025921
TCGA-CJ-6032-01A-11R-1672-07	C2	0.020697373
TCGA-BP-4801-01A-02R-1420-07	C1	0.354378617
TCGA-EU-5904-01A-11R-1672-07	C2	0.388811601
TCGA-B8-5158-01A-01R-1420-07	C1	-0.133599283
TCGA-BP-4969-01A-01R-1334-07	C1	-0.151148366
TCGA-B8-5546-01A-01R-1541-07	C1	-0.078706981
TCGA-B4-5844-01A-11R-1672-07	C2	0.432386916
TCGA-CZ-5452-01A-01R-1503-07	C1	-0.351617997

TCGA-BP-4991-01A-01R-1334-07	C2	0.349208693
TCGA-A3-3316-01A-01R-0864-07	C1	0.022588742
TCGA-B8-A54E-01A-11R-A266-07	C1	-0.440664117
TCGA-BP-5170-01A-01R-1426-07	C1	0.18658452
TCGA-A3-A6NI-01A-11R-A33J-07	C1	-0.291751148
TCGA-B0-5711-01A-11R-1672-07	C2	0.4365423
TCGA-CZ-5451-01A-01R-1503-07	C2	-0.203459123
TCGA-BP-4340-01A-01R-1289-07	C2	-0.176706432
TCGA-BP-5180-01A-01R-1426-07	C3	-0.397033731
TCGA-CJ-4639-01A-02R-1325-07	C2	0.345487349
TCGA-B0-4821-01A-01R-1503-07	C3	-0.288405775
TCGA-CZ-4860-01A-01R-1305-07	C1	0.123151313
TCGA-B0-4813-01A-01R-1277-07	C3	-0.320358017
TCGA-B8-4619-01A-02R-1325-07	C4	-0.090419049
TCGA-CJ-4892-01A-01R-1305-07	C1	0.002828806
TCGA-CJ-4916-01A-01R-1426-07	C1	-0.408271039
TCGA-BP-4964-01A-01R-1334-07	C1	-0.004020557
TCGA-B0-5712-01A-11R-1672-07	C2	-0.29170361
TCGA-BP-4965-01A-01R-1334-07	C2	0.299315425
TCGA-BP-5199-01A-01R-1426-07	C2	0.215410486
TCGA-BP-4973-01A-01R-1334-07	C1	-0.197654161
TCGA-B2-5635-01A-01R-A277-07	C1	0.085371697
TCGA-B0-4844-01A-01R-1277-07	C3	-0.349344264
TCGA-G6-A8L7-01A-11R-A37O-07	C3	-0.337263138
TCGA-B0-5088-01A-01R-1334-07	C1	-0.215387252
TCGA-A3-3324-01A-02R-1325-07	C2	0.220891145
TCGA-BP-4968-01A-01R-1334-07	C1	-0.104698968
TCGA-BP-4352-01A-01R-1289-07	C3	-0.258669686
TCGA-CJ-4872-01A-01R-1305-07	C1	0.362145828
TCGA-DV-A4VZ-01A-11R-A266-07	C1	0.060152924
TCGA-BP-5006-01A-01R-1334-07	C2	0.037654736
TCGA-BP-5009-01A-01R-1334-07	C2	0.036074247
TCGA-B0-4699-01A-01R-1277-07	C1	-0.113513292
TCGA-B0-4834-01A-01R-1305-07	C4	0.126081843
TCGA-BP-4998-01A-01R-1334-07	C2	0.200228098
TCGA-B0-5104-01A-01R-1420-07	C2	0.043526006
TCGA-B0-4945-01A-01R-1420-07	C2	0.234597825
TCGA-B0-4842-01A-02R-1420-07	C3	-0.35398677
TCGA-AK-3447-01A-01R-1766-07	C4	-0.299110794
TCGA-B8-5551-01A-01R-1541-07	C1	-0.177367778
TCGA-A3-3349-01A-01R-1188-07	C2	0.306296184
TCGA-B0-4693-01A-01R-1277-07	C2	0.145875711
TCGA-A3-3346-01A-01R-1766-07	C3	-0.319799644
TCGA-B0-5120-01A-01R-1420-07	C2	0.302620039
TCGA-BP-5177-01A-01R-1426-07	C1	0.325001194
TCGA-B8-4154-01A-01R-1188-07	C1	-0.296264714
TCGA-B8-4153-01B-11R-1672-07	C1	-0.10288484
TCGA-CJ-6030-01A-11R-1672-07	C2	-0.075089295
TCGA-CW-5584-01A-01R-1541-07	C2	0.307770566
TCGA-B0-5696-01A-11R-1541-07	C1	0.076480857
TCGA-BP-4765-01A-01R-1289-07	C2	0.291801404
TCGA-BP-4782-01A-02R-1420-07	C1	-0.204710209

TCGA-B8-5165-01A-01R-1420-07	C2	0.313902107
TCGA-B2-3923-01A-02R-A277-07	C4	-0.199328874
TCGA-AK-3425-01A-02R-1277-07	C1	0.041223609
TCGA-AS-3778-01A-01R-A32Z-07	C2	0.213170979
TCGA-CW-6087-01A-11R-1672-07	C1	-0.066870478
TCGA-B0-5108-01A-01R-1420-07	C2	0.211225922
TCGA-B0-5699-01A-11R-1541-07	C1	0.08704872
TCGA-B0-4814-01A-01R-1277-07	C2	-0.051749321
TCGA-AK-3465-01A-02R-1325-07	C4	-0.217948958
TCGA-CJ-4918-01A-01R-1426-07	C1	0.427097922
TCGA-BP-4344-01A-01R-1289-07	C1	0.404273064
TCGA-B4-5835-01A-11R-1672-07	C1	-0.284545445
TCGA-A3-3372-01A-02R-1325-07	C1	0.24782429
TCGA-BP-4981-01A-01R-1334-07	C3	-0.243822007
TCGA-BP-4170-01A-02R-1289-07	C2	0.206411457
TCGA-CJ-4643-01A-02R-1325-07	C2	0.332165256
TCGA-BP-4977-01A-01R-1334-07	C1	-0.004522698
TCGA-B2-5633-01A-01R-1541-07	C2	0.088571616
TCGA-A3-3311-01A-02R-1325-07	C1	0.024285336
TCGA-BP-5173-01A-01R-1426-07	C1	0.104408154
TCGA-BP-5007-01A-01R-1334-07	C2	-0.098801077
TCGA-A3-3362-01A-02R-1325-07	C2	-0.054801127
TCGA-BP-4781-01A-01R-1305-07	C1	0.333539167
TCGA-A3-3365-01A-01R-0864-07	C2	0.437427904
TCGA-DV-A4W0-01A-11R-A266-07	C1	0.245035909
TCGA-B0-5092-01A-01R-1420-07	C3	-0.43288788
TCGA-CJ-5683-01A-11R-1541-07	C2	-0.031606431
TCGA-BP-4331-01A-01R-1289-07	C1	-0.21017874
TCGA-B2-A4SR-01A-11R-A266-07	C2	0.343285551
TCGA-BP-4976-01A-01R-1334-07	C1	0.214773134
TCGA-B2-4099-01A-02R-1188-07	C1	-0.281701898
TCGA-B2-3923-01B-10R-A277-07	C1	0.215756892
TCGA-G6-A5PC-01A-11R-A33J-07	C3	-0.39200812
TCGA-B0-5812-01A-11R-1672-07	C2	0.523944109
TCGA-BP-4341-01A-01R-1289-07	C2	-0.21228182
TCGA-BP-4795-01A-02R-1420-07	C2	0.429333362
TCGA-AK-3451-01A-02R-1188-07	C3	-0.297564728
TCGA-CZ-5987-01A-11R-1672-07	C3	-0.319437024
TCGA-MM-A564-01A-11R-A266-07	C1	-0.056903704
TCGA-BP-4787-01A-01R-1305-07	C1	0.086903382
TCGA-DV-5566-01A-01R-1541-07	C1	-0.229914184
TCGA-BP-5190-01A-01R-1426-07	C3	-0.466841636
TCGA-BP-4162-01A-02R-1325-07	C2	0.118282544
TCGA-BP-4343-01A-02R-1289-07	C1	0.213240961
TCGA-AK-3458-01A-01R-1503-07	C1	-0.4279208
TCGA-CZ-5457-01A-01R-1503-07	C1	0.076253557
TCGA-A3-A8OX-01A-11R-A37O-07	C2	-0.059191212
TCGA-B0-4817-01A-01R-1277-07	C3	-0.443113085
TCGA-CJ-4888-01A-01R-1305-07	C1	-0.333632043
TCGA-BP-5191-01A-01R-1426-07	C3	-0.45439977
TCGA-B2-5635-01A-01R-1541-07	C1	0.010908653
TCGA-T7-A92I-01A-11R-A37O-07	C1	-0.312733728

TCGA-CJ-4901-01A-01R-1426-07	C1	-0.014567617
TCGA-G6-A8L8-01A-21R-A37O-07	C1	-0.232437609
TCGA-BP-4173-01A-02R-1289-07	C1	-0.048542267
TCGA-AK-3434-01A-02R-1277-07	C1	0.121757345
TCGA-BP-4329-01A-02R-1289-07	C2	0.355056212
TCGA-CZ-5462-01A-01R-1503-07	C1	-0.265505092
TCGA-B0-4706-01A-01R-1503-07	C1	-0.030773247
TCGA-CJ-4920-01A-01R-1426-07	C2	0.202995666
TCGA-B8-A54I-01A-21R-A33J-07	C3	-0.386429402
TCGA-CJ-4893-01A-01R-1305-07	C2	0.270645858
TCGA-BP-4960-01A-01R-1334-07	C1	-0.205122186
TCGA-B2-3924-01A-02R-1325-07	C1	-0.31803264
TCGA-B2-3923-01A-02R-1325-07	C4	-0.178764199
TCGA-BP-5185-01A-01R-1426-07	C3	-0.336930073
TCGA-A3-3357-01A-02R-1420-07	C1	-0.274329331
TCGA-A3-A8CQ-01A-11R-A37O-07	C2	0.011795656
TCGA-BP-5176-01A-01R-1426-07	C1	-0.107097007
TCGA-BP-4160-01A-02R-1289-07	C1	0.017354048
TCGA-B2-3924-01A-02R-A277-07	C1	-0.262910157
TCGA-B0-4822-01A-01R-1277-07	C3	-0.151816914
TCGA-A3-3328-01A-01R-0864-07	C4	-0.239222745
TCGA-CW-5583-01A-02R-1541-07	C2	0.300417574
TCGA-B8-4148-01A-02R-1325-07	C2	-0.162745037
TCGA-BP-4959-01A-01R-1334-07	C1	-0.195680063
TCGA-B0-5097-01A-01R-1420-07	C1	0.337531012
TCGA-CW-6090-01A-11R-1672-07	C2	0.107613756
TCGA-B2-4102-01A-02R-1325-07	C2	-0.000492314
TCGA-BP-4799-01A-01R-1305-07	C1	0.16449649
TCGA-BP-4177-01A-02R-1420-07	C2	0.294451221
TCGA-CZ-4858-01A-01R-1305-07	C3	-0.163566883
TCGA-AK-3450-01A-02R-1277-07	C1	-0.226306305
TCGA-B0-5102-01A-01R-1420-07	C2	-0.270909407
TCGA-BP-4167-01A-02R-1325-07	C1	0.010138803
TCGA-BP-4989-01A-01R-1334-07	C1	-0.176939962
TCGA-BP-5200-01A-01R-1426-07	C2	0.391217538
TCGA-B0-4818-01A-01R-1503-07	C1	0.05894006
TCGA-B0-4714-01A-01R-1277-07	C1	-0.362689184
TCGA-3Z-A93Z-01A-11R-A37O-07	C1	-0.124058212
TCGA-DV-5574-01A-01R-1541-07	C1	0.237832039
TCGA-BP-5194-01A-02R-1426-07	C2	0.236945941
TCGA-BP-4961-01A-01R-1334-07	C2	0.350124886
TCGA-BP-4963-01A-01R-1334-07	C1	-0.18637335
TCGA-AK-3460-01A-02R-1277-07	C2	0.059017297
TCGA-B8-5545-01A-01R-1672-07	C2	0.404370418
TCGA-BP-4165-01A-02R-1289-07	C1	-0.036300931
TCGA-DV-A4VX-01A-11R-A266-07	C3	-0.331794274
TCGA-B0-4846-01A-01R-1277-07	C2	0.233848261
TCGA-CZ-5453-01A-01R-1503-07	C2	-0.077201902
TCGA-B0-5095-01A-01R-1420-07	C1	0.33992707
TCGA-BP-4972-01A-01R-1334-07	C2	0.424207471
TCGA-B0-4712-01A-01R-1503-07	C3	-0.05033214
TCGA-BP-4164-01A-02R-1325-07	C2	0.019348881

TCGA-CZ-5454-01A-01R-1503-07	C1	-0.076908109
TCGA-BP-5202-01A-02R-1426-07	C2	0.073211308
TCGA-CZ-5470-01A-01R-1503-07	C3	-0.336669749
TCGA-CJ-4902-01A-01R-1426-07	C2	0.074199796
TCGA-CJ-4905-01A-02R-1426-07	C2	0.393991194
TCGA-B0-5075-01A-01R-1334-07	C1	-0.257770994
TCGA-BP-4798-01A-01R-1305-07	C1	-0.218404644
TCGA-CJ-4882-01A-02R-1426-07	C1	0.111022792
TCGA-B0-5706-01A-11R-1541-07	C1	0.177269016
TCGA-CJ-4891-01A-01R-1305-07	C3	-0.278023315
TCGA-CZ-5988-01A-11R-1672-07	C1	0.277812358
TCGA-AK-3440-01A-02R-1277-07	C4	-0.251987385
TCGA-AK-3453-01A-02R-1277-07	C3	-0.408301693
TCGA-B0-5697-01A-11R-1541-07	C1	-0.050664107
TCGA-B0-4841-01A-01R-1277-07	C1	0.13870482
TCGA-CJ-5679-01A-11R-1541-07	C3	-0.176549368
TCGA-B0-4845-01A-01R-1277-07	C1	-0.016563356
TCGA-CZ-5465-01A-01R-1503-07	C2	0.003170078
TCGA-CJ-5686-01A-11R-1672-07	C1	-0.086484456
TCGA-BP-4345-01A-01R-1289-07	C1	0.290661324
TCGA-B0-5693-01A-11R-1541-07	C2	0.29999034
TCGA-B0-4838-01A-01R-1305-07	C1	0.103143946
TCGA-B8-5549-01A-01R-1541-07	C2	0.297306119
TCGA-CJ-4876-01A-01R-1305-07	C1	-0.264439483
TCGA-BP-4337-01A-01R-1289-07	C3	-0.100768399
TCGA-BP-4985-01A-01R-1334-07	C1	0.033282711
TCGA-A3-3374-01A-02R-1325-07	C4	-0.245698555
TCGA-A3-3373-01A-02R-1420-07	C2	0.163660119
TCGA-B0-5700-01A-11R-1541-07	C1	-0.262072788
TCGA-B4-5378-01A-01R-1503-07	C2	0.317693718
TCGA-B0-5709-01A-11R-1541-07	C2	0.40737359
TCGA-BP-4351-01A-01R-1289-07	C2	-0.226989176
TCGA-BP-4774-01A-01R-1289-07	C2	0.09384734
TCGA-BP-4986-01A-01R-1334-07	C1	0.202617425
TCGA-B0-5113-01A-01R-1420-07	C2	0.353455109
TCGA-A3-A6NL-01A-11R-A33J-07	C2	0.13373691
TCGA-CJ-5680-01A-11R-1541-07	C1	0.112337992
TCGA-B0-5402-01A-01R-1503-07	C1	0.007599519
TCGA-CZ-4862-01A-01R-1305-07	C2	0.04081692
TCGA-CW-6097-01A-11R-1672-07	C2	0.248497111
TCGA-B4-5843-01A-11R-1672-07	C2	0.189108328
TCGA-A3-3307-01A-01R-0864-07	C2	0.091596453
TCGA-B0-4688-01A-01R-1277-07	C1	-0.1029245
TCGA-CJ-5689-01A-11R-1541-07	C1	0.225929547
TCGA-BP-4335-01A-01R-1289-07	C2	-0.212648702
TCGA-CJ-4908-01A-01R-1426-07	C2	0.393131357
TCGA-CZ-5458-01A-01R-1503-07	C1	-0.060675368
TCGA-B0-4690-01A-01R-1277-07	C1	-0.001555341
TCGA-CJ-4900-01A-01R-1334-07	C1	-0.278427978
TCGA-A3-3359-01A-01R-0864-07	C2	-0.048686667
TCGA-CJ-4907-01A-01R-1426-07	C1	0.371449886
TCGA-CZ-5460-01A-01R-1503-07	C1	-0.194958493

TCGA-B8-4622-01A-02R-1277-07	C1	-0.029399961
TCGA-BP-4994-01A-01R-1334-07	C1	0.121171051
TCGA-B0-4703-01A-01R-1277-07	C1	0.244792886
TCGA-BP-4346-01A-01R-1289-07	C1	-0.163478237
TCGA-B0-4839-01A-01R-1305-07	C1	-0.053553114
TCGA-CZ-5985-01A-11R-1672-07	C1	-0.105380507
TCGA-CZ-4865-01A-02R-1503-07	C1	0.054217466
TCGA-CJ-5675-01A-11R-1541-07	C1	-0.296222749
TCGA-AK-3429-01A-02R-1325-07	C1	-0.107242661
TCGA-B0-4843-01A-01R-1277-07	C3	-0.045412292
TCGA-CJ-4886-01A-01R-1305-07	C2	0.413424065
TCGA-B0-5083-01A-02R-1420-07	C1	-0.141830061
TCGA-B0-5117-01A-01R-1420-07	C4	-0.158568843
TCGA-BP-4325-01A-02R-1289-07	C1	-0.131758276
TCGA-CW-5580-01A-01R-1672-07	C2	0.282478753
TCGA-BP-5000-01A-01R-1334-07	C1	-0.258851413
TCGA-B0-5106-01A-01R-1420-07	C1	0.139058171
TCGA-CJ-6031-01A-11R-1672-07	C1	0.128247231
TCGA-B0-4852-01A-01R-1503-07	C2	0.041959459
TCGA-B0-5690-01A-11R-1541-07	C2	0.379546518
TCGA-B0-4697-01A-01R-1277-07	C3	-0.341201035
TCGA-CZ-4859-01A-02R-1426-07	C2	0.011618046
TCGA-BP-4789-01A-01R-1305-07	C2	0.407534046
TCGA-CZ-5989-01A-11R-1672-07	C1	0.320347389
TCGA-A3-3347-01A-02R-1325-07	C1	0.080483725
TCGA-B0-4827-01A-02R-1420-07	C1	-0.049671354
TCGA-BP-4760-01A-02R-1420-07	C2	0.373409593
TCGA-CW-5581-01A-02R-1541-07	C2	0.112660265
TCGA-AK-3427-01A-01R-0864-07	C4	-0.208297453
TCGA-AK-3445-01A-02R-1277-07	C1	-0.256730153
TCGA-BP-4763-01A-01R-1289-07	C2	0.256785863
TCGA-BP-4776-01A-01R-1289-07	C1	-0.011740297
TCGA-BP-5008-01A-01R-1334-07	C2	0.451181116
TCGA-B0-5119-01A-02R-1420-07	C2	-0.022199425
TCGA-CJ-5684-01A-11R-1541-07	C1	0.155504277
TCGA-BP-4338-01A-01R-1289-07	C3	-0.192660115
TCGA-B0-5085-01A-01R-1334-07	C3	-0.224983405
TCGA-A3-3380-01A-01R-0864-07	C1	0.06329323
TCGA-B0-4691-01A-01R-1277-07	C3	-0.321951011
TCGA-B0-5695-01A-11R-1541-07	C2	0.258855668
TCGA-A3-3317-01A-02R-1325-07	C1	0.281345222
TCGA-CJ-5672-01A-11R-1541-07	C1	-0.335353807
TCGA-CJ-5677-01A-11R-1541-07	C3	0.010502975
TCGA-A3-3367-01A-02R-1420-07	C1	-0.025570908
TCGA-B8-A54K-01A-11R-A33J-07	C2	0.084704361
TCGA-BP-4992-01A-01R-1334-07	C3	-0.33967112
TCGA-B0-5705-01A-11R-1541-07	C1	-0.081009716
TCGA-CW-6088-01A-11R-1672-07	C2	0.370025384
TCGA-BP-4758-01A-01R-1289-07	C1	0.001516829
TCGA-BP-4355-01A-01R-1289-07	C2	0.303459874
TCGA-CZ-4866-01A-01R-1503-07	C1	-0.23832475
TCGA-B8-4621-01A-01R-1503-07	C1	-0.303123518

TCGA-BP-4995-01A-01R-1334-07	C1	0.223123105
TCGA-BP-5004-01A-01R-1334-07	C1	-0.084226852
TCGA-B8-5162-01A-01R-1420-07	C1	0.253249915
TCGA-B0-4847-01A-01R-1277-07	C3	-0.395327303
TCGA-BP-4769-01A-01R-1289-07	C2	0.420820735
TCGA-CZ-5982-01A-11R-1672-07	C2	0.349037355
TCGA-CJ-4870-01A-01R-1305-07	C2	0.176653359
TCGA-AK-3454-01A-02R-1277-07	C3	-0.263524897
TCGA-BP-4975-01A-01R-1334-07	C2	0.149502224
TCGA-CW-5585-01A-01R-1541-07	C2	0.284140196
TCGA-B2-4098-01A-02R-1325-07	C1	-0.499580861
TCGA-CJ-5678-01A-11R-1541-07	C1	-0.325325185
TCGA-B0-5702-01A-11R-1541-07	C3	-0.30012807
TCGA-CJ-4638-01A-02R-1325-07	C3	-0.344985048
TCGA-DV-5573-01A-01R-1541-07	C1	-0.199380663
TCGA-BP-5196-01A-01R-1426-07	C1	-0.094120933
TCGA-A3-3351-01A-02R-1325-07	C2	-0.066448669
TCGA-B8-A54D-01A-21R-A266-07	C3	-0.410717224
TCGA-A3-3322-01A-02R-1325-07	C2	0.143608255
TCGA-B8-A54H-01A-11R-A33J-07	C2	0.111050128
TCGA-A3-A8OW-01A-11R-A37O-07	C1	-0.104481166
TCGA-B0-4701-01A-01R-1277-07	C1	0.0845126
TCGA-B2-4101-01A-02R-1277-07	C1	0.16633619
TCGA-A3-3320-01A-02R-1325-07	C2	0.244009179
TCGA-BP-4999-01A-01R-1334-07	C2	0.368101164
TCGA-BP-4161-01A-02R-1325-07	C1	-0.09211245
TCGA-B8-4143-01A-01R-1188-07	C3	-0.174222323
TCGA-B0-5701-01A-11R-1541-07	C1	-0.338258185
TCGA-BP-4797-01A-01R-1305-07	C1	0.069285683
TCGA-DV-A4W0-05A-11R-A266-07	C2	0.077276863
TCGA-BP-4174-01A-02R-1289-07	C1	-0.117044956
TCGA-A3-3385-01A-02R-1420-07	C1	-0.123509705
TCGA-CZ-5469-01A-01R-1503-07	C3	-0.264078239
TCGA-BP-4970-01A-01R-1334-07	C1	0.316018875
TCGA-BP-4330-01A-01R-1289-07	C2	0.176563633
TCGA-BP-4349-01A-01R-1289-07	C2	0.119500846
TCGA-AK-3461-01A-02R-1277-07	C2	0.242024688
TCGA-A3-3383-01A-02R-1325-07	C1	-0.301894567
TCGA-BP-4159-01A-02R-1289-07	C1	0.152867313
TCGA-B2-5635-01B-04R-A277-07	C1	0.174022771
TCGA-CJ-4889-01A-01R-1305-07	C2	0.327722834
TCGA-BP-4962-01A-01R-1334-07	C1	0.058218399
TCGA-CJ-5682-01A-11R-1541-07	C1	0.434935445
TCGA-BP-4347-01A-01R-1289-07	C2	-0.105170742
TCGA-BP-5186-01A-01R-1426-07	C2	0.293131557
TCGA-CZ-4856-01A-02R-1426-07	C2	0.082217411
TCGA-B0-4824-01A-01R-1277-07	C2	-0.388741167
TCGA-A3-3376-01A-02R-1420-07	C2	0.373908441
TCGA-CJ-4635-01A-02R-1305-07	C1	-0.087166942
TCGA-BP-5183-01A-01R-1426-07	C2	-0.27083941
TCGA-A3-A8OV-01A-11R-A37O-07	C2	-0.361534156
TCGA-B8-5159-01A-01R-1420-07	C2	-0.021059425

TCGA-B8-5163-01A-01R-1420-07	C1	0.234972053
TCGA-CZ-5461-01A-01R-1503-07	C1	0.354078669
TCGA-GK-A6C7-01A-11R-A33J-07	C2	0.355406845
TCGA-BP-4771-01A-01R-1289-07	C1	-0.205384784
TCGA-BP-4803-01A-01R-1305-07	C2	0.271320699
TCGA-B2-5639-01A-01R-1541-07	C2	0.131698977
TCGA-CZ-5455-01A-01R-1503-07	C1	-0.026122917
TCGA-CJ-4897-01A-03R-1426-07	C2	0.286395131
TCGA-B0-5107-01A-01R-1420-07	C3	-0.235288882
TCGA-BP-4759-01A-01R-1289-07	C1	0.01907083
TCGA-B0-4836-01A-01R-1305-07	C1	-0.09182575
TCGA-B8-A7U6-01A-12R-A37O-07	C2	-0.008773259
TCGA-B2-5636-01A-02R-1541-07	C2	0.349813533
TCGA-A3-3382-01A-02R-1325-07	C1	0.163505617
TCGA-CZ-5464-01A-01R-1503-07	C1	-0.143932879
TCGA-CJ-4634-01A-02R-1325-07	C2	0.266063374
TCGA-CJ-6028-01A-11R-1672-07	C2	0.205857802
TCGA-BP-4804-01A-02R-1305-07	C1	0.120340128
TCGA-CJ-4912-01A-01R-1426-07	C3	-0.295503959
TCGA-CJ-4873-01A-01R-1305-07	C3	-0.31619505
TCGA-CJ-4894-01A-01R-1305-07	C2	-0.04640567
TCGA-CW-5588-01A-01R-1541-07	C1	0.21882798
TCGA-A3-3325-01A-01R-0864-07	C1	0.11041097
TCGA-CJ-4871-01A-01R-1305-07	C1	0.310912515
TCGA-CJ-4640-01A-02R-1325-07	C1	-0.188136882
TCGA-BP-4762-01A-02R-1289-07	C2	0.431138913
TCGA-B0-5094-01A-01R-1420-07	C3	-0.283906239
TCGA-CZ-5467-01A-01R-1503-07	C2	0.401218446
TCGA-A3-3363-01A-01R-0864-07	C1	-0.102097376
TCGA-CZ-5468-01A-01R-1503-07	C3	-0.174768389
TCGA-B2-3924-01B-03R-A277-07	C1	0.113269607
TCGA-BP-5181-01A-01R-1426-07	C2	0.173631747
TCGA-B8-5553-01A-01R-1541-07	C2	-0.062388624
TCGA-BP-4761-01A-01R-1289-07	C3	-0.360313181
TCGA-B2-5633-01A-01R-A277-07	C1	-0.001815474
TCGA-A3-3358-01A-01R-1541-07	C1	-0.0541925
TCGA-CZ-4864-01A-01R-1503-07	C2	-0.139058002
TCGA-AK-3436-01A-02R-1325-07	C3	-0.2371751
TCGA-CZ-5466-01A-01R-1503-07	C1	-0.297085105
TCGA-B0-5080-01A-01R-1503-07	C1	0.331103786
TCGA-CZ-4861-01A-01R-1305-07	C2	-0.018708108
TCGA-CJ-5671-01A-11R-1541-07	C1	0.248744346
TCGA-AK-3431-01A-02R-1277-07	C2	0.474822682
TCGA-B0-4811-01A-01R-1503-07	C3	-0.293748251
TCGA-A3-3313-01A-02R-1325-07	C1	-0.155056425
TCGA-B8-5552-01B-11R-1672-07	C2	0.424429832
TCGA-CJ-6027-01A-11R-1672-07	C3	-0.223002798
TCGA-BP-4756-01A-01R-1289-07	C1	-0.353750732
TCGA-A3-3319-01A-02R-1325-07	C3	-0.30148208
TCGA-MW-A4EC-01A-11R-A266-07	C2	-0.254982811
TCGA-B0-4816-01A-01R-1503-07	C2	-0.12361605
TCGA-CZ-4863-01A-01R-1503-07	C1	-0.269751868

TCGA-B0-5110-01A-01R-1420-07	C2	0.379920855
TCGA-CZ-5986-01A-11R-1672-07	C2	0.181348898
TCGA-B0-4828-01A-01R-1277-07	C1	-0.230526325
TCGA-B8-4151-01A-01R-1188-07	C1	-0.19738758
TCGA-EU-5905-01A-11R-1672-07	C1	-0.084605024
TCGA-CJ-4903-01A-01R-1426-07	C1	-0.069835455
TCGA-B0-5698-01A-11R-1672-07	C1	0.016454492
TCGA-B0-4815-01A-01R-1503-07	C1	-0.309996206
TCGA-B0-5081-01A-01R-1334-07	C1	0.164829837
TCGA-A3-3352-01A-01R-0864-07	C1	-0.157795904
TCGA-A3-A6NJ-01A-12R-A33J-07	C2	-0.10103888
TCGA-BP-5184-01A-01R-1426-07	C2	0.149577788
TCGA-CJ-4899-01A-01R-1334-07	C2	0.367383883
TCGA-CJ-4881-01A-01R-1305-07	C3	-0.233895678
TCGA-CJ-4878-01A-01R-1305-07	C2	0.075392076
TCGA-B0-4819-01A-01R-1277-07	C1	-0.164698693
TCGA-B0-4710-01A-01R-1503-07	C2	0.023496063
TCGA-A3-3331-01A-02R-1325-07	C2	0.316026064
TCGA-BP-5174-01A-01R-1426-07	C3	-0.353119718
TCGA-B0-5694-01A-11R-1541-07	C1	-0.037016324
TCGA-B0-5116-01A-02R-1420-07	C2	0.137672351
TCGA-DV-5567-01A-01R-1541-07	C2	0.128665323
TCGA-BP-5169-01A-01R-1426-07	C3	-0.376183516
TCGA-B0-4700-01A-02R-1541-07	C1	-0.187118489
TCGA-AK-3433-01A-02R-1277-07	C4	-0.173024978
TCGA-CJ-4875-01A-01R-1305-07	C3	-0.154781277
TCGA-A3-3335-01A-01R-0864-07	C1	-0.273343975
TCGA-B0-5115-01A-01R-1420-07	C2	0.304264253
TCGA-BP-5195-01A-02R-1426-07	C2	0.357774535
TCGA-BP-4354-01A-02R-1289-07	C1	-0.145656191
TCGA-BP-4974-01A-01R-1334-07	C2	0.348597065
TCGA-CJ-4641-01A-02R-1325-07	C1	-0.215659656
TCGA-B4-5834-01A-11R-1672-07	C2	0.396480326
TCGA-BP-4169-01A-02R-1289-07	C2	0.36680778
TCGA-B2-5633-01B-04R-A277-07	C1	-0.039148577
TCGA-BP-4777-01A-01R-1289-07	C1	-0.338682604
TCGA-B0-5399-01A-01R-1503-07	C1	-0.001544044
TCGA-B0-5099-01A-01R-1420-07	C2	0.122301377
TCGA-BP-4166-01A-02R-1289-07	C1	0.305167094
TCGA-CJ-4890-01A-01R-1305-07	C1	0.273408501
TCGA-B0-5084-01A-01R-1334-07	C1	-0.210292603
TCGA-BP-5168-01A-01R-1420-07	C2	0.064965256
TCGA-BP-4770-01A-01R-1503-07	C1	0.212833442
TCGA-BP-4332-01A-01R-1289-07	C1	0.352530392
TCGA-B4-5832-01A-11R-1672-07	C1	0.293276953
TCGA-B0-4849-01A-01R-1277-07	C2	-0.071655708
TCGA-B8-5164-01A-01R-1420-07	C1	-0.114068981
TCGA-CW-6093-01A-11R-1672-07	C2	0.374399225
TCGA-MM-A84U-01A-11R-A37O-07	C1	-0.250610656
TCGA-CJ-4637-01A-02R-1325-07	C1	-0.15698222
TCGA-CJ-4642-01B-01R-1305-07	C1	-0.355906863
TCGA-BP-5178-01A-01R-1426-07	C3	-0.334742065

TCGA-BP-4790-01A-01R-1305-07	C2	0.376239775
TCGA-B0-5692-01A-11R-1541-07	C1	-0.363532947
TCGA-CJ-4885-01A-01R-1305-07	C2	0.315659939
TCGA-BP-4768-01A-01R-1289-07	C2	0.038215233
TCGA-B0-5713-01A-11R-1672-07	C2	0.138729319
TCGA-CW-5587-01A-01R-1541-07	C2	-0.129734269
TCGA-BP-5201-01A-01R-1426-07	C1	-0.223041068
TCGA-CW-5590-01A-01R-1541-07	C1	0.223843019
TCGA-A3-3370-01A-02R-1420-07	C2	-0.037836527
TCGA-B0-4848-01A-01R-1277-07	C1	0.178165327
TCGA-B0-4698-01A-01R-1503-07	C1	-0.059073148
TCGA-BP-4982-01A-01R-1334-07	C2	0.309364043
TCGA-CJ-4636-01A-02R-1325-07	C1	0.081240131
TCGA-AK-3455-01A-01R-0864-07	C1	-0.374614152
TCGA-A3-A6NN-01A-12R-A33J-07	C2	0.20276434
TCGA-G6-A8L6-01A-11R-A37O-07	C1	-0.089567743
TCGA-B0-5703-01A-11R-1541-07	C2	-0.058317985
TCGA-BP-4176-01A-02R-1289-07	C1	-0.222238606
TCGA-B0-4707-01A-01R-1277-07	C3	-0.233979448
TCGA-CW-5591-01A-01R-1541-07	C1	0.159407622
TCGA-B4-5838-01A-11R-1672-07	C1	0.274610217
TCGA-BP-4775-01A-01R-1289-07	C2	0.02740343
TCGA-BP-4158-01A-02R-1289-07	C2	-0.112283939
TCGA-CJ-5676-01A-11R-1541-07	C1	-0.033129141
TCGA-DV-5565-01A-01R-1541-07	C2	0.338430935
TCGA-BP-4163-01A-02R-1325-07	C1	0.124339165
TCGA-BP-4327-01A-01R-1289-07	C2	-0.06844143
TCGA-BP-5182-01A-01R-1426-07	C1	0.200220338
TCGA-CJ-4884-01A-01R-1305-07	C1	0.078903656
TCGA-CZ-5459-01A-01R-1503-07	C1	-0.397630248
TCGA-B0-5096-01A-01R-1420-07	C1	0.041717891
TCGA-B8-A54J-01A-11R-A33J-07	C2	0.23538513
TCGA-BP-4987-01A-01R-1334-07	C1	0.276236516
TCGA-B2-5641-01A-01R-1541-07	C1	-0.214613441
TCGA-AK-3426-01A-02R-1325-07	C3	-0.384874045
TCGA-B4-5377-01A-01R-1503-07	C2	-0.001166247
TCGA-BP-4342-01A-01R-1289-07	C1	-0.050402112
TCGA-BP-4983-01A-01R-1334-07	C1	0.022386829
TCGA-B0-4833-01A-01R-1305-07	C2	-0.247622788
TCGA-AS-3777-01A-01R-0864-07	C4	-0.305124067
TCGA-CJ-4874-01A-01R-1305-07	C2	0.093327311
TCGA-CJ-4887-01A-01R-1305-07	C1	-0.449240987
TCGA-AK-3428-01A-02R-1277-07	C2	0.318825874
TCGA-B8-A54F-01A-11R-A266-07	C2	-0.133512633
TCGA-BP-5187-01A-01R-1426-07	C2	0.139496191
TCGA-CJ-5681-01A-11R-1541-07	C1	-0.065283892
TCGA-B0-4696-01A-01R-1277-07	C1	0.045891875
TCGA-B0-5710-01A-11R-1672-07	C2	0.443572798
TCGA-BP-5175-01A-01R-1426-07	C3	-0.344306364
TCGA-DV-5569-01A-01R-1541-07	C1	0.106050981
TCGA-BP-5189-01A-02R-1426-07	C1	0.183009728
TCGA-AK-3443-01A-02R-1325-07	C4	-0.251759518

TCGA-CW-5589-01A-01R-1541-07	C2	0.204184548
TCGA-B0-5109-01A-02R-1420-07	C1	0.082518221
TCGA-B8-A8YJ-01A-13R-A39I-07	C1	0.429213137
TCGA-B0-4810-01A-01R-1503-07	C1	-0.078578489
TCGA-BP-5001-01A-01R-1334-07	C3	-0.312219231
TCGA-EU-5906-01A-11R-1672-07	C2	0.308994298
TCGA-BP-4993-01A-02R-1420-07	C2	0.398590814
TCGA-B0-4823-01A-02R-1420-07	C2	0.338161709
TCGA-DV-5576-01A-01R-1541-07	C2	0.127093966
TCGA-MM-A563-01A-11R-A266-07	C1	0.003967629
TCGA-BP-5192-01A-01R-1426-07	C2	0.135648623
TCGA-CJ-4868-01A-01R-1305-07	C1	0.214107051
TCGA-A3-A8OU-01A-11R-A37O-07	C2	-0.167666476
TCGA-B0-4718-01A-01R-1277-07	C2	-0.132917058
TCGA-DV-5568-01A-01R-1541-07	C1	-0.03962779
TCGA-AK-3456-01A-02R-1325-07	C1	-0.229720932
TCGA-B0-4837-01A-01R-1305-07	C3	-0.305716559
TCGA-B0-4694-01A-01R-1277-07	C1	-0.280029402
TCGA-BP-4334-01A-01R-1289-07	C4	-0.325292489
TCGA-B8-4146-01B-11R-1672-07	C1	0.234293476
TCGA-CZ-4854-01A-01R-1305-07	C1	0.02186821
TCGA-DV-5575-01A-01R-1541-07	C2	0.261457417

Table s5

Univariate Cox regression analysis

id	HR	HR.95L	HR.95H	pvalue
age	1.029703838	1.015880474	1.0437153	2.19E-05
grade	2.282634127	1.840530804	2.830932548	5.73E-14
stage	1.925763251	1.676994202	2.211435254	1.60E-20
T	1.972554424	1.660951738	2.3426153	9.65E-15
M	4.499325396	3.25414987	6.220957803	9.22E-20
riskScore	1.676595064	1.453019535	1.93457207	1.48E-12

Table s6

Multivariate Cox regression analysis

id	HR	HR.95L	HR.95H	pvalue
age	1.031258712	1.015924842	1.046824024	5.65E-05
grade	1.458028769	1.142884546	1.860072303	0.002406661
stage	1.734135675	1.100695259	2.732115465	0.017613774
T	0.872786704	0.57516586	1.324412111	0.522516037
M	1.209296605	0.610333217	2.396065362	0.585950378
riskScore	1.29500246	1.099685303	1.525010261	0.001940773

Table s7

SNV of TGF-β pathway genes across cancer types

	ACC	BLCA	BRCA	CESC	CHOL
ACVR1	0.010869565	0.009708738	0.002028398	0.003460208	0
ACVR1C	0	0.007281553	0.004056795	0.01384083	0
ACVR2A	0	0.004854369	0.010141988	0.031141869	0.039215686
ACVR2B	0.010869565	0.004854369	0.005070994	0.01384083	0
ACVRL1	0.010869565	0.014563107	0.001014199	0.01384083	0
AMH	0	0.002427184	0	0.003460208	0
AMHR2	0.010869565	0.012135922	0.003042596	0.017301038	0
BMP2	0	0.002427184	0.003042596	0.003460208	0.039215686
BMP4	0	0.009708738	0.003042596	0.017301038	0.019607843
BMP5	0	0.024271845	0.003042596	0.006920415	0.019607843
BMP6	0.010869565	0.02184466	0.002028398	0.006920415	0.019607843
BMP7	0.010869565	0.004854369	0	0.006920415	0.019607843
BMP8A	0.02173913	0.004854369	0.001014199	0	0
BMP8B	0.010869565	0.009708738	0.001014199	0.006920415	0
BMPR1A	0.02173913	0.007281553	0.003042596	0.031141869	0
BMPR1B	0.010869565	0.009708738	0.009127789	0.01384083	0
BMPR2	0	0.031553398	0.00811359	0.041522491	0
CDKN2B	0.010869565	0.007281553	0	0.003460208	0
CHRD	0.02173913	0.026699029	0.005070994	0.027681661	0
COMP	0	0.031553398	0.006085193	0.017301038	0
CREBBP	0.010869565	0.111650485	0.017241379	0.089965398	0
CUL1	0	0.048543689	0.004056795	0.034602076	0
DCN	0.010869565	0.009708738	0.004056795	0.01384083	0
E2F4	0	0.002427184	0.001014199	0.006920415	0
E2F5	0	0.004854369	0.001014199	0.006920415	0
EP300	0.02173913	0.13592233	0.017241379	0.121107266	0.039215686
FST	0	0.012135922	0.004056795	0.006920415	0.019607843
GDF5	0.010869565	0.019417476	0.002028398	0.017301038	0
GDF6	0	0.007281553	0.005070994	0.017301038	0
GDF7	0	0.002427184	0.002028398	0.006920415	0
ID1	0	0.033980583	0.003042596	0.017301038	0
ID2	0	0.009708738	0.001014199	0.010380623	0
ID3	0	0.004854369	0.002028398	0.003460208	0
ID4	0	0	0	0.006920415	0.019607843
IFNG	0	0.004854369	0.003042596	0.010380623	0
INHBA	0.010869565	0.014563107	0.006085193	0.027681661	0.039215686
INHBB	0.010869565	0.014563107	0.001014199	0.017301038	0
INHBC	0	0	0.001014199	0.003460208	0
INHBE	0	0.012135922	0.002028398	0.006920415	0
LEFTY1	0	0	0.001014199	0.006920415	0
LEFTY2	0	0.007281553	0	0.01384083	0.019607843
LTBP1	0.010869565	0.046116505	0.013184584	0.017301038	0
MAPK1	0.010869565	0.007281553	0.001014199	0.044982699	0.019607843

MAPK3	0	0.02184466	0.002028398	0.006920415	0
MYC	0	0.009708738	0.002028398	0.024221453	0
NODAL	0	0.009708738	0	0.010380623	0
NOG	0.010869565	0.007281553	0	0.010380623	0
PITX2	0.032608696	0.012135922	0.005070994	0.020761246	0
PPP2CA	0	0.007281553	0.003042596	0.01384083	0
PPP2CB	0	0.004854369	0.003042596	0.003460208	0
PPP2R1A	0	0.016990291	0.007099391	0.031141869	0
PPP2R1B	0	0.016990291	0.002028398	0.017301038	0
RBL1	0	0.029126214	0.013184584	0.031141869	0
RBL2	0	0.029126214	0.002028398	0.017301038	0
RBX1	0	0.002427184	0	0.010380623	0
RHOA	0	0.041262136	0.006085193	0.020761246	0.019607843
ROCK1	0.02173913	0.016990291	0.011156187	0.044982699	0.019607843
ROCK2	0	0.029126214	0.009127789	0.024221453	0
RPS6KB1	0	0.004854369	0.007099391	0.01384083	0
RPS6KB2	0	0.004854369	0.007099391	0.006920415	0
SKP1	0	0.012135922	0.001014199	0.003460208	0
SMAD1	0.010869565	0.007281553	0.004056795	0.010380623	0
SMAD2	0	0.012135922	0.009127789	0.01384083	0
SMAD3	0	0.024271845	0.004056795	0.027681661	0
SMAD4	0	0.019417476	0.009127789	0.051903114	0.019607843
SMAD5	0	0	0.004056795	0.024221453	0.019607843
SMAD6	0	0.007281553	0.002028398	0.010380623	0
SMAD7	0.010869565	0.012135922	0.001014199	0.006920415	0
SMAD9	0	0.012135922	0.006085193	0.003460208	0
SMURF1	0	0.016990291	0.014198783	0.031141869	0
SMURF2	0	0.019417476	0.005070994	0.010380623	0.019607843
SP1	0	0.026699029	0.006085193	0.027681661	0.019607843
TFDP1	0.010869565	0.007281553	0.004056795	0.010380623	0
TGFB1	0	0.002427184	0.002028398	0	0
TGFB2	0.02173913	0.002427184	0.002028398	0.010380623	0
TGFB3	0.010869565	0.007281553	0.006085193	0.017301038	0
TGFBR1	0.010869565	0.02184466	0.006085193	0.020761246	0
TGFBR2	0.02173913	0.02184466	0.009127789	0.038062284	0.058823529
THBS1	0.010869565	0.02184466	0.011156187	0.017301038	0
THBS2	0.02173913	0.036407767	0.007099391	0.017301038	0
THBS3	0	0.014563107	0.005070994	0.020761246	0
THBS4	0.010869565	0.016990291	0.009127789	0.010380623	0
TNF	0	0.012135922	0	0.003460208	0
ZFYVE16	0.010869565	0.029126214	0.010141988	0.01384083	0.019607843
ZFYVE9	0.010869565	0.063106796	0.004056795	0.010380623	0

COAD	DLBC	ESCA	GBM	HNSC	KICH
0.01754386	0	0.005434783	0.007692308	0.007905138	0
0.025062657	0	0.016304348	0.01025641	0.017786561	0
0.100250627	0	0.02173913	0.007692308	0.001976285	0.015151515
0.045112782	0	0.010869565	0.002564103	0.003952569	0
0.030075188	0	0.010869565	0.01025641	0.009881423	0
0.007518797	0	0.005434783	0	0	0.015151515
0.025062657	0	0.005434783	0.012820513	0.001976285	0
0.022556391	0.027027027	0.010869565	0.002564103	0.007905138	0
0.020050125	0	0.005434783	0.002564103	0.007905138	0
0.027568922	0.054054054	0.005434783	0.017948718	0.009881423	0
0.025062657	0	0.010869565	0.007692308	0.007905138	0
0.032581454	0.027027027	0.010869565	0.002564103	0.005928854	0.015151515
0.022556391	0.027027027	0.005434783	0.002564103	0	0
0.012531328	0	0.010869565	0.002564103	0.007905138	0
0.020050125	0	0.02173913	0.002564103	0.001976285	0
0.035087719	0	0.016304348	0.007692308	0.009881423	0
0.102756892	0	0.016304348	0.01025641	0.009881423	0
0.010025063	0	0	0.002564103	0	0
0.045112782	0	0.043478261	0.002564103	0.019762846	0.015151515
0.032581454	0.027027027	0.010869565	0.002564103	0	0
0.125313283	0.135135135	0.048913043	0.017948718	0.061264822	0
0.042606516	0.027027027	0.010869565	0.020512821	0.019762846	0
0.025062657	0	0.016304348	0.01025641	0.005928854	0
0.020050125	0	0	0.002564103	0.003952569	0.015151515
0.025062657	0	0.016304348	0	0.007905138	0
0.07518797	0.027027027	0.065217391	0.01025641	0.071146245	0
0.037593985	0.027027027	0.016304348	0.007692308	0.001976285	0
0.047619048	0	0.010869565	0.007692308	0.009881423	0.015151515
0.037593985	0	0.010869565	0.002564103	0.009881423	0
0.015037594	0	0.005434783	0.002564103	0.001976285	0
0.020050125	0	0.005434783	0	0.001976285	0
0	0	0.005434783	0	0.001976285	0
0.005012531	0.054054054	0.005434783	0	0.001976285	0
0	0	0.010869565	0	0	0
0.015037594	0	0.010869565	0.005128205	0.001976285	0
0.052631579	0	0.043478261	0.012820513	0.007905138	0
0.010025063	0	0.027173913	0.002564103	0.005928854	0
0.020050125	0	0	0.002564103	0.003952569	0
0.01754386	0	0.016304348	0.002564103	0.001976285	0
0.015037594	0	0	0.005128205	0.009881423	0
0.012531328	0	0.005434783	0.01025641	0.005928854	0
0.067669173	0	0.038043478	0.007692308	0.039525692	0
0.010025063	0	0.005434783	0.007692308	0.011857708	0

0.012531328	0	0.010869565	0.01025641	0.001976285	0
0.022556391	0.108108108	0.010869565	0	0.011857708	0
0.007518797	0	0	0	0.001976285	0
0.052631579	0	0.016304348	0	0.001976285	0
0.012531328	0.027027027	0.010869565	0.007692308	0.001976285	0
0.010025063	0.054054054	0.005434783	0.005128205	0	0
0.012531328	0	0	0.002564103	0.001976285	0
0.040100251	0	0.027173913	0.005128205	0.013833992	0
0.020050125	0	0.005434783	0.01025641	0.007905138	0
0.040100251	0	0.010869565	0.017948718	0.029644269	0
0.047619048	0	0.016304348	0.007692308	0.009881423	0
0	0	0	0	0.009881423	0.015151515
0.015037594	0.054054054	0.016304348	0.002564103	0.015810277	0
0.077694236	0	0.048913043	0.01025641	0.029644269	0
0.037593985	0	0.027173913	0.007692308	0.02173913	0
0.022556391	0	0.016304348	0.005128205	0.011857708	0
0.020050125	0	0.005434783	0	0.005928854	0
0.005012531	0.054054054	0	0.005128205	0.003952569	0
0.022556391	0	0	0.007692308	0.003952569	0
0.050125313	0	0.005434783	0.002564103	0.005928854	0.015151515
0.052631579	0	0.02173913	0.002564103	0.007905138	0.015151515
0.120300752	0	0.065217391	0.005128205	0.023715415	0
0.01754386	0.027027027	0.027173913	0	0.003952569	0
0.020050125	0	0.016304348	0	0.001976285	0
0.015037594	0.027027027	0.010869565	0.005128205	0	0
0.040100251	0.027027027	0.010869565	0.002564103	0.011857708	0
0.067669173	0.027027027	0.010869565	0.007692308	0.011857708	0.015151515
0.022556391	0	0.02173913	0.007692308	0.009881423	0
0.035087719	0	0.016304348	0.007692308	0.007905138	0
0.035087719	0	0.010869565	0.005128205	0.007905138	0
0.015037594	0	0.005434783	0.007692308	0.005928854	0
0.040100251	0	0.005434783	0.01025641	0.007905138	0
0.020050125	0	0.010869565	0	0.001976285	0
0.040100251	0	0.027173913	0.002564103	0.005928854	0
0.050125313	0	0.059782609	0.005128205	0.037549407	0
0.062656642	0	0.005434783	0.015384615	0.011857708	0
0.095238095	0.027027027	0.027173913	0.005128205	0.019762846	0
0.032581454	0	0.010869565	0.007692308	0.011857708	0
0.032581454	0.027027027	0.010869565	0.005128205	0.015810277	0
0.005012531	0.054054054	0	0.005128205	0	0
0.05764411	0	0.010869565	0.007692308	0.013833992	0
0.050125313	0	0.016304348	0.012820513	0.02173913	0

KIRC	KIRP	LAML	LGG	LIHC	LUAD
0.005952381	0	0	0.001976285	0.002747253	0.010695187
0	0	0	0.001976285	0.002747253	0.021390374
0.00297619	0.017793594	0.007462687	0.003952569	0.035714286	0.012477718
0.00297619	0.003558719	0	0	0.005494505	0.005347594
0.005952381	0	0	0.005928854	0.008241758	0.023172906
0	0.003558719	0	0	0	0.001782531
0.00297619	0.007117438	0	0.003952569	0.005494505	0.012477718
0	0	0	0	0.002747253	0.001782531
0.00297619	0.003558719	0	0.001976285	0.002747253	0.008912656
0.005952381	0.007117438	0	0.003952569	0.013736264	0.010695187
0.00297619	0.003558719	0.007462687	0.001976285	0.002747253	0.01426025
0	0.007117438	0	0	0.005494505	0.026737968
0	0.003558719	0	0	0	0.003565062
0.00297619	0.003558719	0	0	0	0
0.005952381	0.010676157	0	0	0.005494505	0.010695187
0.00297619	0.003558719	0	0.001976285	0.005494505	0.017825312
0.005952381	0.007117438	0.007462687	0	0.010989011	0.016042781
0	0.003558719	0	0.003952569	0	0.003565062
0.008928571	0.007117438	0	0.001976285	0.005494505	0.028520499
0.00297619	0	0	0.001976285	0.010989011	0.007130125
0.011904762	0.028469751	0.007462687	0.007905138	0.019230769	0.042780749
0.005952381	0.007117438	0.014925373	0.001976285	0.005494505	0.021390374
0.00297619	0	0	0.001976285	0.013736264	0.012477718
0	0.007117438	0	0	0.002747253	0.008912656
0	0.003558719	0	0.001976285	0.002747253	0
0.020833333	0.042704626	0.007462687	0.003952569	0.027472527	0.017825312
0	0	0	0.005928854	0.002747253	0.019607843
0.008928571	0.010676157	0.007462687	0.001976285	0.008241758	0.019607843
0	0.003558719	0	0.003952569	0	0.026737968
0	0.003558719	0	0.001976285	0	0.007130125
0	0	0	0	0.010989011	0.001782531
0	0	0	0	0.002747253	0.007130125
0	0	0	0.001976285	0.005494505	0.005347594
0	0	0	0	0	0.001782531
0	0.007117438	0	0	0.010989011	0.010695187
0.00297619	0	0	0.003952569	0.005494505	0.055258467
0	0.003558719	0.007462687	0.001976285	0.002747253	0.010695187
0	0.003558719	0	0	0	0.012477718
0	0.003558719	0	0.001976285	0.008241758	0.010695187
0	0.007117438	0	0	0.005494505	0.005347594
0.00297619	0.003558719	0	0.003952569	0.002747253	0.007130125
0.011904762	0.014234875	0.007462687	0.009881423	0.03021978	0.099821747
0	0.003558719	0	0.003952569	0.008241758	0.007130125

0.00297619	0	0	0.001976285	0	0.008912656
0.00297619	0	0.007462687	0	0.002747253	0.005347594
0	0.003558719	0.007462687	0.001976285	0.005494505	0.005347594
0	0.007117438	0	0	0.002747253	0
0.005952381	0	0	0.003952569	0.008241758	0.016042781
0	0	0	0.003952569	0.005494505	0.003565062
0.00297619	0.003558719	0	0.001976285	0.002747253	0.003565062
0	0.007117438	0	0	0.008241758	0.017825312
0.011904762	0	0.007462687	0.001976285	0.013736264	0.007130125
0.00297619	0	0	0.007905138	0.016483516	0.01426025
0.00297619	0.010676157	0	0.005928854	0.024725275	0.01426025
0	0.003558719	0	0.001976285	0	0
0	0	0	0.001976285	0.002747253	0.007130125
0.023809524	0.007117438	0	0.001976285	0.016483516	0.032085561
0.008928571	0.003558719	0.007462687	0.003952569	0.008241758	0.010695187
0.008928571	0	0	0.003952569	0.005494505	0.007130125
0	0	0.007462687	0.001976285	0.005494505	0.005347594
0	0	0	0.001976285	0.002747253	0.007130125
0	0.003558719	0	0.003952569	0	0.012477718
0.005952381	0	0	0	0	0.016042781
0	0.003558719	0.007462687	0.003952569	0.008241758	0.012477718
0.00297619	0.003558719	0.007462687	0	0.010989011	0.033868093
0	0	0	0	0.008241758	0.003565062
0	0	0	0.001976285	0.002747253	0.003565062
0.005952381	0.003558719	0	0.003952569	0.002747253	0.001782531
0.00297619	0.007117438	0.007462687	0.005928854	0.005494505	0.008912656
0.008928571	0	0	0.001976285	0.005494505	0.012477718
0.00297619	0.003558719	0	0.003952569	0.002747253	0.010695187
0.00297619	0	0	0	0.008241758	0.010695187
0	0	0.014925373	0.001976285	0.002747253	0.016042781
0	0	0	0	0.013736264	0.003565062
0	0	0	0.001976285	0.010989011	0.017825312
0.00297619	0	0.007462687	0.001976285	0	0.001782531
0	0.003558719	0	0	0.002747253	0.008912656
0	0.003558719	0	0.003952569	0.002747253	0.010695187
0.020833333	0.021352313	0.007462687	0.009881423	0.013736264	0.017825312
0.005952381	0.007117438	0	0.003952569	0.019230769	0.071301248
0.011904762	0.007117438	0	0.001976285	0.013736264	0.003565062
0.00297619	0.007117438	0.007462687	0.005928854	0	0.021390374
0.00297619	0.007117438	0	0.001976285	0.002747253	0.003565062
0.00297619	0.007117438	0	0	0.008241758	0.012477718
0.00297619	0.010676157	0	0.005928854	0.010989011	0.023172906

LUSC	MESO	OV	PAAD	PCPG	PRAD
0.012219959	0.0125	0.004587156	0	0	0
0.020366599	0	0.006880734	0.006329114	0	0.004132231
0.00814664	0	0.004587156	0.006329114	0	0.002066116
0.00610998	0	0.002293578	0.006329114	0	0
0.018329939	0	0.004587156	0.006329114	0	0.006198347
0	0	0	0	0	0
0.016293279	0	0.004587156	0.006329114	0	0.002066116
0.00407332	0	0.002293578	0.006329114	0	0.002066116
0.020366599	0	0.002293578	0.006329114	0	0.004132231
0.032586558	0	0.004587156	0.006329114	0	0.006198347
0.014256619	0	0.002293578	0	0	0
0.00814664	0	0.004587156	0.012658228	0	0.004132231
0.00203666	0	0.004587156	0.006329114	0	0
0.00610998	0	0.002293578	0	0	0
0.014256619	0	0.004587156	0.012658228	0	0.004132231
0.00407332	0	0.004587156	0.006329114	0	0.002066116
0.020366599	0	0.009174312	0.006329114	0	0.004132231
0.00203666	0	0.002293578	0	0	0.002066116
0.036659878	0	0.009174312	0.006329114	0	0.002066116
0.012219959	0	0.002293578	0.006329114	0	0.006198347
0.073319756	0.025	0.032110092	0.018987342	0	0.010330579
0.020366599	0	0.004587156	0.006329114	0	0.002066116
0.018329939	0.0125	0.004587156	0	0	0.004132231
0.00203666	0	0.004587156	0.006329114	0	0
0.00610998	0	0.004587156	0.006329114	0	0
0.038696538	0.0125	0.016055046	0.012658228	0	0.008264463
0.00203666	0	0.004587156	0.006329114	0	0.002066116
0.020366599	0	0.002293578	0.006329114	0	0
0.018329939	0	0.006880734	0.006329114	0	0.004132231
0.00203666	0	0.004587156	0.006329114	0	0
0.00407332	0	0.002293578	0	0	0
0	0	0	0	0	0
0.00610998	0	0.002293578	0	0	0
0.00407332	0.0125	0.002293578	0	0	0
0.00407332	0.0125	0	0	0	0
0.038696538	0	0.006880734	0.006329114	0	0.002066116
0.00407332	0	0	0.006329114	0	0
0.012219959	0	0	0.006329114	0	0
0.00610998	0	0.002293578	0	0	0.002066116
0.00610998	0	0.002293578	0	0.005617978	0
0.010183299	0	0	0.006329114	0	0
0.067209776	0	0.013761468	0	0.005617978	0.006198347
0.010183299	0	0.002293578	0	0	0.002066116

0.00407332	0	0	0	0	0
0.00610998	0.0125	0.006880734	0	0	0.004132231
0.010183299	0	0.004587156	0	0	0.002066116
0.00203666	0	0.002293578	0	0	0
0.016293279	0	0.004587156	0	0	0.002066116
0.00610998	0	0.006880734	0.012658228	0	0.004132231
0.00203666	0	0	0.006329114	0	0.002066116
0.024439919	0	0.009174312	0.006329114	0	0.002066116
0.016293279	0	0.002293578	0.018987342	0	0.002066116
0.026476578	0	0.016055046	0.012658228	0	0.008264463
0.026476578	0.0125	0.009174312	0.006329114	0.005617978	0.006198347
0.00203666	0	0.002293578	0	0	0
0.00610998	0	0.004587156	0.006329114	0	0.004132231
0.028513238	0.025	0.002293578	0.025316456	0	0.006198347
0.018329939	0	0.004587156	0.006329114	0	0.002066116
0.00407332	0	0.004587156	0	0	0
0.016293279	0.0125	0.004587156	0.012658228	0	0.004132231
0.00203666	0	0	0.006329114	0	0.002066116
0.016293279	0	0	0	0	0
0.00814664	0	0.002293578	0.006329114	0	0.004132231
0.00407332	0	0	0.006329114	0	0.002066116
0.022403259	0	0.004587156	0.170886076	0	0.008264463
0.00407332	0	0	0	0	0.002066116
0.00407332	0	0	0	0	0.002066116
0.00610998	0	0.006880734	0.006329114	0	0.004132231
0.012219959	0.0125	0	0.006329114	0	0.004132231
0.00610998	0.025	0.006880734	0	0	0.002066116
0.00407332	0	0	0	0	0.004132231
0.014256619	0	0.006880734	0.006329114	0	0.004132231
0.010183299	0	0.004587156	0.012658228	0	0.004132231
0.00407332	0	0	0.006329114	0	0.004132231
0.018329939	0.0125	0.004587156	0.006329114	0	0.002066116
0.00814664	0	0	0	0	0.002066116
0.016293279	0	0.004587156	0.006329114	0	0.004132231
0.026476578	0.0125	0.01146789	0.037974684	0	0
0.016293279	0	0.018348624	0.018987342	0.005617978	0.002066116
0.071283096	0	0.013761468	0.006329114	0.005617978	0.004132231
0.026476578	0	0.006880734	0.006329114	0	0.004132231
0.010183299	0.0125	0.004587156	0.012658228	0	0.006198347
0.00814664	0.0125	0.006880734	0	0	0
0.020366599	0	0	0.012658228	0	0.006198347
0.028513238	0.0125	0.009174312	0.012658228	0	0

READ	SARC	SKCM	STAD	TGCT	THCA
0.014705882	0	0.027837259	0.011547344	0	0
0.022058824	0	0.04496788	0.018475751	0	0
0.044117647	0.004219409	0.014989293	0.108545035	0.006896552	0
0.014705882	0.008438819	0.019271949	0.032332564	0	0.002053388
0	0	0.053533191	0.018475751	0	0
0	0	0	0.006928406	0.006896552	0
0.007352941	0.004219409	0.057815846	0.025404157	0.006896552	0
0.022058824	0	0.040685225	0.020785219	0	0
0	0.004219409	0.019271949	0.025404157	0.006896552	0
0.022058824	0.012658228	0.113490364	0.034642032	0	0
0	0.012658228	0.038543897	0.032332564	0	0
0.014705882	0.004219409	0.038543897	0.023094688	0	0
0	0.012658228	0.012847966	0.002309469	0	0
0	0	0.010706638	0.013856813	0	0
0.014705882	0.004219409	0.010706638	0.030023095	0	0
0.022058824	0	0.019271949	0.030023095	0	0
0.036764706	0.008438819	0.032119914	0.060046189	0	0
0	0	0.002141328	0.002309469	0	0
0.029411765	0.012658228	0.066381156	0.030023095	0	0
0.007352941	0.008438819	0.027837259	0.020785219	0	0
0.066176471	0.008438819	0.098501071	0.092378753	0.013793103	0
0.022058824	0.008438819	0.023554604	0.041570439	0	0.002053388
0.014705882	0	0.047109208	0.020785219	0	0
0.007352941	0.004219409	0.010706638	0.006928406	0	0
0.007352941	0	0.010706638	0.013856813	0	0
0.044117647	0.016877637	0.064239829	0.055427252	0	0
0.022058824	0.004219409	0.023554604	0.018475751	0	0
0.022058824	0.004219409	0.087794433	0.036951501	0	0
0.014705882	0.008438819	0.012847966	0.043879908	0	0
0	0.004219409	0.002141328	0.013856813	0	0
0	0.004219409	0.00856531	0.009237875	0	0
0	0	0.006423983	0.004618938	0	0.002053388
0	0.004219409	0.006423983	0.002309469	0	0
0	0.012658228	0	0.002309469	0	0.002053388
0.007352941	0.004219409	0.019271949	0.006928406	0	0
0.029411765	0.008438819	0.074946467	0.07852194	0.006896552	0
0.014705882	0.008438819	0.03640257	0.023094688	0	0
0.014705882	0.004219409	0.042826552	0.016166282	0	0.002053388
0	0	0.025695931	0.004618938	0	0
0	0	0.021413276	0.006928406	0	0
0	0	0.021413276	0.016166282	0	0
0.029411765	0.012658228	0.137044968	0.057736721	0	0.004106776
0.007352941	0.004219409	0.010706638	0.013856813	0	0

0	0.008438819	0.032119914	0.011547344	0	0
0.007352941	0.008438819	0.017130621	0.011547344	0	0
0.007352941	0	0.00856531	0.009237875	0	0
0.014705882	0	0.004282655	0.006928406	0	0
0	0	0.027837259	0.020785219	0	0.004106776
0.007352941	0	0.00856531	0.013856813	0	0
0.007352941	0	0.004282655	0.009237875	0	0
0.014705882	0.008438819	0.025695931	0.030023095	0	0.004106776
0.014705882	0.004219409	0.019271949	0.027713626	0	0
0.022058824	0	0.038543897	0.032332564	0	0.004106776
0.014705882	0	0.019271949	0.025404157	0	0
0.007352941	0	0.006423983	0.002309469	0	0
0.007352941	0.004219409	0.021413276	0.046189376	0	0
0.058823529	0.008438819	0.027837259	0.057736721	0	0
0.036764706	0.004219409	0.038543897	0.043879908	0.006896552	0
0	0.008438819	0.010706638	0.018475751	0	0
0	0	0.010706638	0.018475751	0	0
0	0	0.006423983	0.004618938	0	0
0.014705882	0	0.017130621	0.009237875	0	0
0.051470588	0.004219409	0.021413276	0.020785219	0	0
0.036764706	0.016877637	0.014989293	0.018475751	0	0
0.176470588	0.004219409	0.010706638	0.07852194	0	0
0.022058824	0.004219409	0.004282655	0.006928406	0	0
0	0	0.023554604	0.009237875	0	0
0.014705882	0	0.00856531	0.016166282	0	0
0.022058824	0	0.03640257	0.055427252	0	0
0.036764706	0.004219409	0.019271949	0.053117783	0.006896552	0
0.022058824	0.008438819	0.029978587	0.057736721	0	0.002053388
0.014705882	0.012658228	0.019271949	0.020785219	0.006896552	0.002053388
0.007352941	0.008438819	0.014989293	0.025404157	0	0
0	0.008438819	0.004282655	0.006928406	0	0
0.058823529	0	0.023554604	0.043879908	0	0.002053388
0.007352941	0	0.021413276	0.018475751	0	0
0.029411765	0.012658228	0.014989293	0.027713626	0	0
0	0.008438819	0.029978587	0.034642032	0	0.002053388
0.014705882	0.012658228	0.113490364	0.03926097	0	0
0.066176471	0	0.077087794	0.046189376	0	0.004106776
0.029411765	0.008438819	0.038543897	0.03926097	0.006896552	0.002053388
0.022058824	0.012658228	0.092077088	0.050808314	0	0
0	0.004219409	0.017130621	0.004618938	0	0
0.022058824	0	0.025695931	0.032332564	0	0.002053388
0.044117647	0.016877637	0.03640257	0.030023095	0	0

THYM	UCEC	UCS	UVM
0	0.085066163	0.01754386	0
0	0.052930057	0	0
0.008196721	0.162570888	0	0
0	0.11342155	0	0
0	0.066162571	0	0.0125
0.008196721	0.011342155	0	0
0.008196721	0.052930057	0	0
0.008196721	0.081285444	0	0
0	0.092627599	0.01754386	0
0.008196721	0.079395085	0	0
0	0.043478261	0.01754386	0
0	0.088846881	0	0
0	0.030245747	0	0
0	0.02268431	0	0
0	0.064272212	0.01754386	0
0.008196721	0.034026465	0	0
0	0.088846881	0	0
0	0.013232514	0.01754386	0
0	0.06805293	0	0
0	0.041587902	0	0
0	0.147448015	0.052631579	0
0	0.069943289	0	0
0.008196721	0.060491493	0	0
0.008196721	0.028355388	0	0
0	0.062381853	0	0
0.016393443	0.160680529	0.01754386	0.0125
0.008196721	0.051039698	0.01754386	0
0.008196721	0.043478261	0	0
0	0.066162571	0.01754386	0
0	0.030245747	0	0
0.008196721	0.017013233	0	0
0.008196721	0.026465028	0	0
0	0.020793951	0	0
0	0.020793951	0	0
0	0.035916824	0	0
0	0.124763705	0.01754386	0
0	0.041587902	0	0
0.008196721	0.026465028	0	0
0	0.037807183	0	0
0	0.035916824	0	0
0	0.026465028	0	0
0.008196721	0.105860113	0.01754386	0
0	0.100189036	0	0

0	0.034026465	0	0
0	0.060491493	0.035087719	0
0	0.02268431	0	0
0	0.037807183	0	0
0	0.075614367	0	0
0	0.049149338	0	0
0	0.04536862	0	0
0.008196721	0.173913043	0.280701754	0.025
0	0.058601134	0	0.0125
0	0.096408318	0	0.0125
0	0.088846881	0	0
0	0.015122873	0	0
0.008196721	0.056710775	0	0
0.008196721	0.143667297	0	0
0.008196721	0.11342155	0.052631579	0
0	0.096408318	0	0
0	0.037807183	0	0
0	0.020793951	0	0
0	0.032136106	0	0
0	0.066162571	0	0
0	0.102079395	0	0
0	0.096408318	0.01754386	0
0	0.128544423	0	0
0	0.041587902	0	0
0	0.079395085	0	0
0	0.037807183	0	0
0	0.139886578	0	0
0.008196721	0.107750473	0	0
0.008196721	0.107750473	0.01754386	0
0	0.098298677	0	0
0	0.024574669	0.01754386	0
0	0.079395085	0	0
0	0.049149338	0	0
0	0.085066163	0	0
0.008196721	0.081285444	0	0
0	0.088846881	0	0.0125
0	0.128544423	0.035087719	0.0125
0.008196721	0.069943289	0	0.0125
0	0.071833648	0	0
0	0.017013233	0	0
0	0.120982987	0.035087719	0
0.008196721	0.102079395	0.01754386	0

Table s8

TGF-β pathway genes expression alterations across cancer types

	BLCA	CHOL	COAD	ESCA	KICH
CREBBP	-0.225754898	1.358463043	NA	NA	0.218466604
THBS3	NA	2.77614054	NA	NA	-0.693414697
NODAL	NA	2.832988235	2.340346413	NA	NA
FST	NA	-0.181342589	-0.719788484	NA	-0.044261387
INHBA	2.67734005	NA	5.410218664	2.676634159	-0.235129677
TGFBR1	-0.139788841	1.712104521	0.390152152	NA	NA
PPP2R1A	0.29922326	2.205716454	0.170560586	0.29269037	-0.284894509
MAPK1	NA	1.272218394	-0.21193425	NA	NA
E2F5	1.134780545	3.381193447	1.656281617	NA	-0.692877463
CDKN2B	NA	1.775618114	-3.186177822	NA	NA
SMAD7	-0.96232688	1.71780602	-0.820365166	NA	-0.266778446
AMH	3.105120346	5.563781916	4.133541457	1.98578537	3.077531349
ACVR2B	1.148742088	0.712487355	1.004781706	NA	-0.669939092
TGFB1	NA	2.774757111	NA	0.959350326	-0.415609348
SMURF1	NA	2.288860353	0.485351515	0.699616623	1.117300446
GDF6	-0.033382888	NA	-1.079713531	NA	1.319517232
ROCK1	-0.991267431	1.447647266	-0.3541301	NA	NA
BMP8B	1.814489278	4.610009641	-0.712473518	0.977182163	-1.553911055
ZFYVE16	NA	1.362593762	NA	NA	-0.83376201
LEFTY1	NA	3.430438771	0.044510071	NA	-3.435367412
SKP1	-0.240627499	1.679393161	-0.17755153	NA	-0.461030894
BMP5	-3.27502908	-1.373190669	-2.392719472	0.233065836	-1.590997548
BMPR1A	-0.734450996	1.914699233	-0.315098199	NA	-1.2125847
E2F4	0.590235598	2.197727723	0.705920578	0.817236725	0.322818743
RBX1	0.510594228	1.691924355	0.199240816	0.592012962	-0.256440008
LTBP1	-0.677353205	2.098658017	NA	NA	-2.381260032
INHBC	NA	-3.705331298	NA	-0.005355237	-0.634232302
TFDP1	1.063109314	1.516547076	1.08715913	1.144189199	-1.010907807
BMPR2	-0.403035895	1.448786443	-0.069140074	NA	-0.762268991
SMAD4	-0.347850226	0.921788748	-0.439882537	NA	-0.178474979
GDF7	NA	NA	-0.747004057	-1.997783756	-3.739917654
ACVR2A	NA	1.146948471	-0.434920284	NA	-0.966016725
COMP	NA	4.853267786	6.010189308	NA	-3.086439061
PPP2R1B	0.616139016	-1.07050234	0.172594502	NA	NA
DCN	-2.451572387	NA	-1.685039296	NA	-3.687334366
SP1	NA	2.206209063	NA	0.443510948	-0.478850416
ID3	NA	1.864671279	-0.589286546	NA	-0.721048075
CUL1	NA	0.490679202	0.519445575	0.524745907	NA
ID4	-0.706990429	2.934886671	0.02780238	-0.654721727	-2.866352322
ACVR1	NA	1.987584924	0.324199213	0.837578894	NA
CHRD	-0.85883333	-1.284707968	NA	NA	-3.378145641
SMURF2	NA	2.551615442	0.454268715	NA	-1.070171166
TGFBR2	-1.189420471	0.882963309	-0.202636423	-0.93568193	-0.928004915
PPP2CA	NA	1.373391708	0.107809104	0.493487899	-0.325208993

PITX2	-1.767105514	4.483175772	NA	NA	-3.053651653
THBS2	NA	3.304403133	2.326755001	1.575055183	-0.808857541
SMAD2	NA	1.998281564	-0.151801431	NA	NA
THBS4	NA	3.098900326	-1.62497202	NA	2.115679235
BMPR1B	NA	NA	NA	NA	1.898532803
SMAD9	-1.34024334	1.160405015	-0.274850077	-0.79131522	-2.798769713
NOG	1.679781975	NA	-0.391837051	NA	-0.455077855
PPP2CB	-0.595115899	0.767048435	-0.645016466	NA	-0.74217045
SMAD3	NA	2.695870924	-0.222886297	0.694365495	0.316723119
RBL1	1.357610628	3.117801001	1.562646196	1.652956505	-0.297492885
MAPK3	NA	2.371679216	-0.899514466	NA	-0.550311502
INHBE	NA	-1.050861377	1.371765373	3.728434651	NA
TGFB2	-0.274865947	5.429636603	0.74179879	1.656456022	-1.730140835
RPS6KB2	0.650249763	1.215463026	0.432331352	NA	NA
ACVR1C	NA	-1.91979322	-1.13825446	NA	3.623699236
SMAD6	NA	1.726675005	0.993237905	NA	-0.886285034
BMP4	-0.536242327	2.479322929	1.954067811	NA	-2.516799969
TGFB3	-1.1741218	2.143861472	NA	NA	-0.834892604
LEFTY2	-0.26283636	NA	0.291366411	-0.846869551	-2.339401291
RHOA	NA	1.274550989	NA	NA	-0.465089892
ZFYVE9	-0.31416493	1.014745611	-0.33890499	NA	-0.859964253
EP300	NA	1.435774104	-0.23426333	NA	NA
ACVRL1	-0.899286427	2.208537611	-1.504257768	NA	-1.316658622
RBL2	-0.403396369	NA	-0.057068265	NA	NA
GDF5	NA	NA	-1.32754215	NA	-1.698346909
RPS6KB1	NA	1.323568245	0.369367937	0.459970888	-0.650316639
MYC	-1.439930513	NA	2.117023816	0.636158469	-1.784666009
ROCK2	-1.013871684	0.977527876	NA	NA	-1.006071374
IFNG	NA	NA	0.951991752	1.290141699	-0.2821425
TNF	NA	3.031482092	NA	2.084938259	-1.198397516
INHBB	-0.952005591	NA	2.400991649	NA	1.374194037
BMP7	-0.728552576	3.487623199	3.418024994	NA	-0.750254046
SMAD5	-0.185614024	2.203479207	0.582576788	NA	-0.282139275
BMP6	0.034119251	3.036809045	-2.079722777	NA	-3.200583121
BMP2	-0.86429582	2.663114489	-1.79576421	NA	-0.829905303
ID2	-0.956220688	-0.720727065	-0.566385465	NA	-2.011354844
SMAD1	NA	NA	-0.493495458	0.629665069	NA
THBS1	-2.36321566	NA	NA	NA	-2.859834981
ID1	NA	NA	NA	NA	-3.515091458
AMHR2	-0.940507546	-1.984925975	-1.33937496	-1.74267619	NA
BMP8A	1.47438433	3.206832745	0.613974172	1.756620314	-0.759082284

KIRC	KIRP	LIHC	LUAD	LUSC
0.219292288	NA	0.529267366	NA	NA
1.253417092	1.251706067	1.508656332	0.769708411	NA
1.63668742	NA	2.309644269	NA	NA
0.197575838	-1.246784036	NA	1.07121704	3.776516772
0.891411801	-0.066568663	-0.330874637	-0.290766955	-0.128338752
NA	-0.19700332	0.791240498	NA	NA
-0.182350367	NA	1.127281004	0.268368299	0.476913192
0.167080633	-0.421640149	0.78628496	-0.05561557	NA
-0.181010442	0.282234041	1.899621312	1.407450044	0.824136924
1.695625258	0.515806093	1.974918479	-1.144212664	-0.430286599
NA	-0.450685189	NA	-0.980648891	-1.468299366
2.712002615	2.971320651	3.293922622	1.820031629	2.840005042
-0.290075068	NA	0.437577917	0.774887527	0.642349376
1.561311126	NA	1.3035984	-0.364488472	-0.057342151
0.286970373	0.491473482	1.096027009	0.482438409	0.252074642
3.584166972	-1.048238776	-0.325960225	1.139841617	NA
NA	-0.419478769	0.476316103	-0.229908162	-0.460421367
1.234342685	1.788806167	2.126089627	1.380993407	1.114745175
-0.357798522	-0.493510679	0.572739237	NA	-0.569678752
0.778411646	NA	NA	NA	0.250921595
-0.329943767	-0.38939529	0.864068943	NA	-0.055303014
-1.273250651	-3.45644834	-1.726274257	-0.737158308	-2.387518983
-0.42591777	-0.152070423	0.772915023	-0.338040796	-0.308869984
0.417050645	0.372776761	1.206904216	0.47743698	0.70568899
0.305547135	0.300459643	0.940076413	NA	0.439922789
-0.966555831	-2.110753843	0.102546015	NA	0.640115591
NA	NA	-0.734276488	1.365134594	1.674155144
-0.231095528	-0.180113691	0.854254612	0.976022691	1.596503749
-0.160047981	-0.469832004	NA	-1.200032836	-1.362190135
-0.224598903	-0.26215357	0.404901628	-0.250886815	-0.160319914
-1.456012493	-0.890915542	NA	-0.178963662	-0.38398443
-0.436985585	-0.250264721	NA	NA	0.379328172
0.422024542	-0.551335563	3.563985065	3.056279901	NA
-0.371714778	-0.764607632	0.475008651	0.680274564	0.571830159
-2.551065409	-2.865177156	-1.667203762	-1.336083987	-1.295248148
0.252557594	0.639147578	0.7226635	NA	0.220263492
0.61255805	-0.279710845	NA	-1.058503608	-0.788585032
0.284977798	0.282238143	0.54121914	0.160245829	0.456335338
-0.403702811	NA	0.026149301	-1.675346986	-1.5676118
NA	0.290840542	NA	0.50493893	0.511269934
0.678698122	-0.326221514	0.807203181	1.386949141	0.131681264
NA	0.440173284	1.052863355	NA	-0.489433095
-0.231335849	-1.159679108	NA	-1.553523141	-2.677190237
-0.076756839	-0.109571929	0.618295699	NA	0.15483928

1.926219642	2.019874325	5.918061638	8.292906024	8.304853932
1.396069612	1.74781254	NA	3.060913305	2.101190351
-0.218263742	-0.229953711	1.054220342	0.010193482	NA
2.30080863	0.031984226	6.373337243	NA	NA
-2.182343358	-3.229590066	-0.180648807	0.216259499	-0.061109015
0.613585912	1.180837206	0.300871264	-1.284469241	-1.851257083
2.704805639	-0.264616582	0.282277597	0.446835579	-0.584650739
-0.471914125	-0.149039821	-0.162358453	-0.69316496	-0.723567807
-0.214227499	NA	1.09350753	NA	0.795853119
0.425989843	0.401231856	2.007746957	1.097723485	1.545580235
0.330702903	NA	1.37850532	-0.323348297	-0.690522588
3.373963306	3.207975497	NA	2.299537582	2.786334647
-0.402473875	-1.036912133	2.008394888	-0.909763597	-1.009761123
0.778424631	0.162419765	0.885510837	0.800770919	0.950169624
-0.729459362	-1.271961361	-1.021992743	NA	2.807410766
-0.396353988	-0.768810555	-0.288471548	-2.491079049	-2.597875052
-0.008066604	NA	2.426482049	0.084098823	-0.610006011
0.991640657	NA	NA	NA	NA
-0.25505457	-1.395328506	NA	-1.846326091	-3.306987527
-0.21166558	0.214809504	0.857072877	-0.417985216	-0.814897998
-0.774960498	-1.257448413	0.389511065	-1.160849448	-0.483331683
NA	-0.445769766	0.582749724	NA	NA
0.83709608	-0.892637169	1.321845461	-2.47148486	-3.019680111
-0.37330979	-0.387694938	-0.13692568	-0.288919388	-0.528958514
-0.737942277	2.16807597	2.933366823	0.306577285	-1.19431812
0.347494204	0.377860769	0.733481865	0.647358375	0.536608918
1.519239974	1.052152439	-0.564770058	NA	0.635364738
-0.130444742	-0.51182726	0.824126545	-0.049284466	-0.487237765
4.520772568	NA	NA	1.455736429	NA
NA	NA	NA	-0.245799745	-0.392977365
3.817266256	0.833531299	NA	1.204452347	NA
-3.139795863	-0.638315319	4.736981462	0.99512718	4.299750612
NA	-0.176111742	0.899014197	0.31288153	0.22552458
-2.139548413	-1.439929876	0.326422946	-0.492757273	-1.541019395
0.879487389	NA	0.888897287	-1.610508762	-2.181194057
0.526318711	NA	-0.81237419	-0.802170432	-1.178121235
-0.388506002	-0.585823225	NA	0.604580791	0.679333889
-0.348586037	-1.384141993	-1.01657065	-0.616203009	-1.521710908
-0.7764768	NA	-1.206755303	-0.890368946	-0.0705338
0.941572125	1.487481283	-2.131462848	0.062123635	-1.79190612
1.442842689	NA	1.776506562	2.504238563	1.807037724

PAAD	PCPG	PRAD	READ	SARC	SKCM
NA	NA	NA	NA	NA	NA
NA	1.806032894	0.601832246	NA	NA	NA
NA	NA	-0.2877999	NA	NA	NA
NA	-1.091390431	-0.868584152	NA	NA	NA
NA	NA	0.700104882	4.040172224	NA	NA
NA	1.358367001	-0.263378977	NA	1.738652571	NA
NA	-0.864578883	0.145762521	NA	NA	NA
NA	NA	-0.15846364	NA	NA	NA
NA	NA	1.013085654	1.482498808	NA	NA
NA	NA	-0.449934212	-2.128940406	NA	NA
-0.714747706	NA	-0.406408925	NA	NA	NA
NA	4.19952058	3.258064695	NA	NA	NA
NA	NA	0.378712678	NA	NA	NA
NA	NA	NA	NA	NA	NA
NA	-1.275140019	NA	NA	NA	NA
NA	3.647986505	-0.51765705	-2.755379486	NA	NA
NA	NA	-0.299789902	-0.977362596	NA	NA
NA	3.831955729	NA	NA	NA	NA
NA	NA	NA	NA	NA	NA
NA	1.894687059	-1.034338304	NA	NA	NA
NA	NA	-0.136643642	NA	-0.893050383	NA
NA	NA	-2.27661987	-2.249350881	NA	NA
NA	NA	-0.41223227	-1.070063147	NA	NA
NA	NA	-0.075413822	1.004628678	NA	NA
NA	NA	0.243181906	NA	NA	NA
NA	NA	-0.53939113	NA	NA	NA
NA	NA	-1.184719531	NA	NA	NA
NA	NA	NA	1.04581847	NA	NA
NA	NA	-0.273377328	NA	NA	NA
NA	NA	-0.175720228	-0.801910374	NA	NA
NA	NA	-0.867164261	NA	NA	NA
NA	0.943843181	-0.247657214	NA	NA	NA
NA	NA	2.087686899	5.486859536	NA	NA
-0.382387091	NA	0.376971938	NA	NA	NA
NA	-3.877691332	-1.008167724	-2.674968587	NA	NA
NA	-0.518804266	-0.147515143	NA	NA	NA
NA	NA	-1.140237987	NA	NA	NA
NA	NA	-0.204763913	NA	NA	NA
NA	NA	-1.530259341	NA	NA	NA
NA	1.340678666	NA	NA	NA	NA
NA	-1.740133221	1.146755316	NA	NA	NA
NA	-0.46834449	0.180534121	NA	NA	NA
NA	-1.224134521	-0.748357662	NA	NA	NA
NA	-0.490918333	NA	NA	NA	NA

NA	NA	-1.195300781	6.317188849	NA	NA
NA	NA	0.927994777	NA	NA	NA
NA	NA	-0.179769476	-0.651272175	NA	NA
NA	NA	1.588322135	-3.860588286	NA	NA
NA	3.55736235	0.684192137	NA	NA	NA
NA	2.861622557	-0.483212063	NA	NA	NA
NA	NA	-1.055308736	NA	NA	NA
NA	NA	-0.35434138	-0.733194653	NA	NA
NA	NA	-0.53118124	NA	NA	NA
NA	0.894182501	-0.276426614	1.570616583	2.239829579	NA
NA	0.856868299	-0.121107498	NA	NA	NA
NA	2.936084836	-0.480169852	NA	NA	NA
NA	NA	-0.870000882	NA	NA	NA
NA	-0.419676745	0.413444085	NA	NA	NA
NA	NA	-0.533583044	NA	NA	NA
NA	NA	-0.728301829	1.563643471	NA	NA
NA	-2.157412379	-0.751841308	2.490011348	NA	NA
-1.01716857	NA	-1.236630108	NA	NA	NA
NA	-3.415555348	NA	NA	NA	NA
NA	-0.391242892	-0.141471068	NA	NA	NA
NA	NA	NA	-0.858353486	NA	NA
NA	NA	NA	NA	NA	NA
NA	NA	-0.811432665	-1.240277218	NA	NA
NA	NA	-0.296152212	-0.574994232	NA	NA
NA	NA	-0.767655806	-2.187222315	NA	NA
NA	-0.578142551	0.2090789	NA	NA	NA
NA	-1.905808427	0.759250721	1.500387444	NA	NA
NA	NA	-0.619525457	NA	NA	NA
NA	NA	NA	NA	NA	NA
NA	NA	NA	NA	NA	NA
NA	NA	0.505450453	NA	NA	NA
NA	5.635045415	-0.904147879	NA	NA	NA
NA	NA	-0.211103012	NA	NA	NA
NA	NA	0.943619355	-1.979398007	NA	NA
NA	NA	-0.849199632	-2.259338354	NA	NA
NA	-1.719838009	-0.792786876	NA	NA	NA
NA	NA	-0.404750612	NA	NA	NA
NA	NA	NA	-1.876381362	NA	NA
NA	NA	-1.376609713	NA	NA	NA
NA	-4.512771419	1.406376759	NA	NA	NA
NA	NA	0.745980596	NA	NA	NA

STAD	THCA	THYM	UCEC
0.234905066	-0.375948574	NA	-0.615180451
0.445772276	-0.195863615	NA	-0.453977785
NA	NA	NA	NA
0.739916078	0.376405093	NA	-1.04221361
4.151977833	0.796035158	NA	NA
0.516297311	1.608750929	NA	-0.806755975
NA	0.330746141	0.532142531	0.434298704
0.309363572	0.272752811	NA	-0.299067148
1.140228944	NA	NA	0.854229761
NA	2.847500034	NA	NA
NA	-0.272130485	NA	-0.51099216
3.35132158	0.985597391	NA	4.682312184
0.945549909	NA	NA	-0.26999165
NA	1.39910727	NA	-0.432045737
0.785928252	NA	NA	-0.31333515
-0.245112825	3.307377991	NA	-2.268813113
0.335875527	NA	-0.848494861	-1.098129078
0.51619816	-0.570117571	NA	NA
0.576182726	-0.282090934	NA	-0.379670021
5.698386154	NA	NA	6.833344901
NA	-0.136748853	NA	-0.518825332
-1.185357917	-1.783989091	-3.132153247	4.317938256
NA	-0.652738336	NA	-0.562148729
0.512164446	NA	NA	NA
0.150237781	NA	NA	0.769529143
NA	1.175992863	NA	-0.885063029
1.772157793	-1.095590674	NA	-0.313089045
1.021728678	-0.380198826	NA	0.537387721
0.509482374	NA	NA	-1.084039135
NA	-0.388411478	NA	-0.870863571
-1.032125278	NA	NA	-1.627108356
NA	NA	NA	-0.575630909
4.807724969	4.396639028	NA	1.123345265
0.614384841	-0.479800626	-0.934973972	0.939658322
-1.19106702	-1.994409754	NA	-4.515572245
0.559678564	NA	NA	-0.420211986
-1.001427864	-1.045431054	NA	-0.396913436
0.399935469	NA	NA	NA
-1.200426444	-1.38090306	NA	-1.278085928
0.793441402	0.677265568	NA	-0.357334702
NA	-0.173787976	-3.450727781	-1.678078138
0.520614959	0.317730861	NA	-0.243515135
NA	-0.246393493	NA	-2.411739725
0.234605072	-0.141329983	NA	NA

NA	0.799671747	NA	6.335315338
2.831531135	NA	NA	-2.023642394
0.237328509	-0.33065606	NA	-0.255195971
NA	NA	NA	-1.735277543
NA	-1.511084495	NA	1.241493467
-0.980263443	-1.561347952	NA	-0.533762092
NA	NA	NA	3.133904176
-0.171449668	-0.398623871	NA	-0.912848898
0.44625582	-0.136927146	NA	-1.286246402
1.629100523	-0.318410183	NA	0.527722615
-0.805287171	NA	NA	-1.017986468
1.580190552	0.951802671	NA	NA
0.912570573	0.98295988	NA	-0.959832027
NA	NA	NA	1.105983238
NA	NA	NA	NA
0.922004423	NA	NA	NA
NA	0.290976764	NA	0.745419459
0.89769007	0.114722189	NA	-1.449953974
NA	0.668852164	NA	-3.880677202
NA	0.044815885	NA	-0.263409148
NA	-0.626738818	NA	-0.731570457
0.423675787	-0.319880274	NA	NA
NA	NA	NA	-1.344475325
NA	-0.386522733	-1.074187433	-1.051067191
NA	NA	NA	3.879095749
0.54423675	-0.23659609	NA	-0.321159843
NA	-0.801476961	NA	-1.677776538
0.640331025	-0.375922715	NA	-1.37397015
2.065836774	-0.953320909	NA	2.289327884
NA	-0.704892597	NA	2.331042307
2.016424916	1.703329394	NA	NA
NA	-0.559197435	NA	NA
0.454443646	-0.27867937	NA	-0.918288545
-1.481650465	NA	NA	-1.080597091
NA	-2.065844771	NA	0.330737895
NA	0.224187941	NA	NA
0.318874997	-0.644449885	NA	-0.973174889
0.268271313	0.372647771	NA	-2.684210355
-0.889207331	-0.645426028	NA	0.713592173
1.603779671	NA	NA	-3.953050102
2.195899778	-2.217449774	NA	-0.835902583

Table s9

Survival landscape of TGF-β pathway genes across cancer types

	ACC	BLCA	CHOL	COAD	DLBC
PPP2CB	NA	1.038122248	NA	0.931876735	NA
SMAD1	NA	1.146239292	NA	NA	NA
INHBE	NA	NA	NA	4.022749573	NA
TGFB3	1.062271084	1.027963751	NA	1.069153119	NA
NOG	NA	1.033124427	NA	5.218511467	NA
SMAD6	NA	0.822028548	NA	NA	NA
AMH	1.187987928	NA	NA	NA	NA
ROCK1	NA	NA	NA	NA	NA
GDF5	NA	NA	NA	NA	NA
MYC	1.040354411	NA	NA	NA	NA
RBL1	1.439017975	NA	NA	NA	NA
RBX1	1.074627175	NA	NA	NA	NA
ACVR1	NA	1.045711373	NA	NA	NA
CUL1	NA	NA	NA	NA	NA
BMP8A	NA	NA	NA	NA	NA
LTBP1	1.104033699	1.013367007	NA	NA	NA
MAPK3	0.939628713	1.030942704	NA	NA	NA
RPS6KB2	1.182287211	NA	NA	NA	NA
BMP8B	NA	NA	NA	NA	NA
ID1	NA	0.998663175	NA	NA	NA
PITX2	4.423395637	NA	NA	NA	NA
SMURF1	NA	NA	NA	1.087432268	NA
BMPR2	NA	NA	NA	NA	NA
BMP7	NA	NA	NA	NA	NA
SMURF2	1.277154541	NA	1.283368052	NA	NA
E2F5	NA	NA	NA	NA	NA
SKP1	NA	NA	NA	NA	NA
CHRD	NA	NA	NA	2.156364962	NA
THBS2	1.043112615	NA	NA	NA	NA
ZFYVE16	NA	NA	NA	NA	NA
ACVR1C	NA	NA	NA	NA	5537202.482
BMPR1A	NA	NA	NA	NA	NA
LEFTY1	3.766786293	NA	NA	NA	NA
BMP2	NA	NA	NA	NA	NA
RHOA	1.007130862	NA	NA	NA	NA
ACVR2A	NA	NA	NA	NA	NA
MAPK1	1.082763802	NA	NA	NA	NA
GDF6	NA	NA	NA	4.133587158	NA
ACVR2B	NA	NA	NA	NA	NA
BMP6	NA	NA	NA	1.539403416	NA
ID2	NA	0.986284635	NA	NA	NA
AMHR2	NA	NA	NA	NA	61569549.37
TGFB2	1.401458486	NA	NA	1.284094775	NA

SMAD7	NA	NA	NA	NA	NA
COMP	NA	1.001316458	NA	NA	NA
GDF7	NA	NA	NA	NA	NA
FST	NA	NA	NA	1.108677235	NA
BMPR1B	NA	NA	NA	NA	NA
TGFB1	NA	NA	NA	NA	NA
ID3	NA	NA	NA	NA	NA
LEFTY2	NA	NA	NA	NA	NA
ROCK2	1.302842853	NA	NA	NA	NA
SMAD2	1.504938888	NA	NA	NA	NA
RPS6KB1	NA	NA	NA	NA	NA
RBL2	0.890214273	NA	NA	NA	NA
THBS4	1.058412705	NA	NA	NA	NA
TFDP1	1.156742358	NA	NA	NA	NA
SP1	1.092044897	NA	NA	NA	NA
BMP5	2.154521954	NA	NA	NA	NA
TGFBR1	1.1942978	NA	NA	NA	NA
NODAL	NA	NA	NA	NA	NA
SMAD9	NA	NA	NA	1.060719427	NA
PPP2CA	NA	NA	1.135907674	NA	NA
SMAD5	NA	NA	NA	NA	NA
SMAD4	1.388465888	NA	NA	NA	NA
CDKN2B	NA	NA	NA	NA	NA
E2F4	NA	NA	NA	NA	NA
PPP2R1A	NA	NA	NA	NA	NA
ID4	NA	NA	NA	NA	NA
ZFYVE9	NA	NA	NA	NA	NA
TNF	NA	NA	NA	NA	NA
THBS1	NA	1.003925338	NA	1.010301222	NA
SMAD3	1.145197184	NA	NA	1.105410958	NA
INHBC	NA	NA	NA	232.2144002	NA
THBS3	1.099112784	1.018318678	NA	1.16749553	NA
ACVRL1	1.058904192	1.053947071	NA	NA	NA
EP300	NA	NA	NA	NA	NA
INHBA	2.088337699	NA	NA	1.052485671	NA
INHBB	NA	1.051818611	NA	NA	NA
TGFBR2	NA	1.012072935	NA	NA	NA
BMP4	NA	NA	NA	NA	NA
CREBBP	NA	NA	NA	NA	NA
PPP2R1B	1.170924499	NA	NA	NA	NA
DCN	NA	1.005271026	NA	NA	NA
IFNG	NA	0.806590366	NA	NA	NA

ESCA	KICH	KIRC	KIRP	LAML	LIHC
NA	NA	0.951395653	NA	NA	1.042363402
NA	NA	NA	NA	NA	NA
NA	1.440496468	1.071205986	1.045451139	NA	NA
NA	1.290751029	1.026284933	1.44913855	NA	NA
NA	NA	NA	NA	NA	5.866892302
NA	NA	0.719268065	NA	1.827639771	NA
NA	1.384375858	1.261406276	1.196391006	NA	NA
NA	NA	0.890748699	NA	NA	NA
NA	NA	2.725758883	NA	NA	NA
NA	NA	NA	1.011344203	NA	NA
NA	2.079273732	NA	2.232196696	NA	1.513518397
NA	NA	1.018892142	NA	NA	1.042652946
NA	NA	NA	1.064646246	NA	1.043779381
NA	1.194236286	NA	NA	0.963155052	1.042386222
NA	NA	1.357494088	NA	NA	NA
NA	1.121258537	NA	1.097570634	NA	NA
NA	NA	0.958106663	NA	1.067009259	1.062130529
NA	1.54250708	1.171223001	NA	NA	NA
NA	NA	NA	NA	1.132780857	NA
NA	NA	NA	NA	NA	NA
NA	31495173.28	1.029300124	NA	NA	1.309715584
NA	NA	NA	NA	NA	NA
NA	NA	0.933052015	NA	NA	NA
NA	NA	NA	NA	5.237950122	NA
NA	NA	NA	NA	0.835319415	1.183030789
NA	NA	1.461760769	1.88423843	NA	1.421309812
1.114540265	NA	0.951319697	NA	NA	1.043362397
NA	NA	1.15478309	1.1009042	NA	NA
NA	1.071755554	1.00526858	1.018173699	NA	NA
NA	1.661776948	0.873310636	NA	0.808039082	NA
NA	NA	NA	NA	NA	NA
NA	NA	0.789507663	NA	NA	1.396437429
NA	NA	NA	NA	NA	NA
NA	NA	0.951405871	NA	2.620314061	NA
NA	1.024863689	0.992423064	0.992684457	1.004928828	1.007754893
NA	NA	0.589093025	NA	NA	1.331508172
NA	NA	0.94583866	NA	NA	1.083660851
NA	NA	0.922040279	NA	NA	1.428820871
NA	NA	0.48844931	NA	NA	1.730457765
NA	NA	0.916989206	NA	NA	NA
NA	NA	0.992443569	NA	NA	NA
NA	NA	NA	3.524108198	NA	2.492945232
NA	NA	NA	1.368770401	NA	1.036869332

NA	0.827711449	NA	NA	NA	1.083284694
NA	NA	1.029194574	1.069704	NA	NA
NA	NA	0.622543741	NA	NA	NA
NA	NA	NA	NA	NA	1.002695476
NA	NA	NA	NA	NA	NA
NA	NA	1.008139988	1.028788996	1.009363675	1.010532398
NA	NA	NA	NA	NA	NA
NA	NA	NA	1.355174804	NA	1.15846213
NA	NA	0.897878367	1.160385097	NA	NA
NA	NA	0.677954248	NA	NA	1.72637381
NA	1.894772385	NA	1.458000706	0.85153337	NA
NA	NA	0.943129455	NA	NA	NA
NA	NA	1.006219627	1.370648258	NA	NA
NA	1.09823856	0.937094856	NA	NA	1.035467719
NA	NA	NA	NA	NA	NA
1.116663821	NA	0.658714945	1.672193666	NA	NA
NA	NA	NA	1.064240458	NA	NA
NA	NA	3.556202236	NA	NA	NA
NA	NA	0.935013883	NA	0.3807503	NA
1.049218632	1.146796888	0.974668575	NA	NA	1.065958221
NA	1.516809347	0.895953389	NA	NA	NA
NA	NA	0.828777497	0.764756675	0.886468645	NA
NA	NA	0.878061464	1.310412452	NA	1.169984381
NA	0.860429988	1.115786373	1.091334877	1.028288725	1.074955549
NA	NA	NA	NA	1.069241733	NA
NA	NA	0.977966698	NA	NA	NA
NA	NA	0.798132439	NA	0.881035734	1.203369601
NA	NA	NA	NA	0.926664412	NA
NA	NA	0.994804887	NA	NA	NA
NA	NA	NA	1.075949513	1.077716063	NA
NA	100.7527472	NA	NA	NA	NA
NA	NA	1.08139587	NA	NA	NA
NA	NA	0.956662554	NA	NA	NA
NA	NA	0.920798583	NA	NA	NA
NA	NA	NA	1.249549632	NA	NA
NA	NA	NA	NA	NA	NA
NA	NA	0.987881036	NA	NA	NA
NA	NA	0.94904266	NA	0.291023282	NA
NA	NA	0.862542549	NA	NA	NA
NA	NA	0.878015578	NA	NA	0.978499893
NA	1.05290745	1.011216334	1.050495967	NA	NA
NA	NA	1.230444733	NA	1.377027218	NA

LUAD	LUSC	MESO	OV	PAAD	PCPG
NA	NA	NA	NA	NA	NA
NA	NA	NA	NA	NA	NA
NA	NA	NA	NA	NA	NA
NA	NA	NA	1.051388453	NA	NA
NA	NA	NA	NA	1.183947383	NA
NA	NA	NA	NA	NA	NA
NA	0.8902906	1.184560525	NA	NA	1.295323694
NA	NA	NA	NA	NA	NA
NA	NA	1.02995469	1.104862664	NA	NA
1.006807766	NA	NA	NA	NA	NA
NA	NA	1.631038227	NA	NA	NA
NA	NA	NA	NA	NA	0.715615411
NA	NA	NA	NA	NA	NA
NA	NA	NA	NA	NA	NA
NA	NA	NA	NA	NA	NA
1.021860267	NA	NA	1.013793108	NA	NA
NA	1.03151585	0.931092794	NA	NA	NA
NA	NA	NA	NA	NA	NA
NA	NA	1.691546387	NA	NA	NA
1.002642353	NA	NA	NA	NA	NA
NA	NA	1.893692515	NA	1.911495264	NA
NA	NA	NA	NA	NA	NA
NA	NA	NA	NA	NA	NA
1.032297181	NA	NA	NA	0.849659779	NA
NA	NA	1.110253531	0.918070175	NA	NA
NA	NA	NA	NA	NA	NA
NA	NA	0.9465233	NA	NA	NA
NA	NA	NA	NA	0.714248488	NA
NA	NA	1.003363992	1.005301718	NA	NA
NA	NA	NA	NA	NA	NA
NA	NA	NA	NA	NA	NA
NA	NA	NA	NA	NA	NA
NA	NA	NA	0.952446921	NA	NA
NA	1.030882829	NA	NA	NA	NA
NA	NA	1.004732304	0.996901602	NA	NA
NA	NA	NA	NA	NA	NA
NA	NA	NA	NA	NA	NA
NA	NA	1.154507641	NA	NA	NA
NA	NA	NA	NA	NA	NA
NA	NA	NA	NA	NA	NA
NA	NA	NA	NA	NA	NA
1.718222673	NA	NA	NA	NA	NA
1.048453853	NA	NA	NA	NA	NA

NA	1.033729998	NA	NA	NA	NA
NA	NA	1.001721561	NA	NA	1.019459205
NA	NA	NA	NA	0.24498457	NA
1.013407529	NA	NA	NA	NA	NA
NA	NA	NA	NA	NA	NA
NA	NA	1.009365529	NA	NA	NA
1.008058198	NA	NA	1.006093982	NA	NA
NA	NA	NA	NA	0.148280371	NA
NA	NA	NA	NA	NA	NA
1.158372879	NA	NA	NA	NA	NA
NA	NA	NA	NA	NA	NA
NA	NA	0.916281482	NA	NA	NA
NA	NA	NA	NA	NA	NA
1.001090316	NA	NA	NA	NA	NA
1.039122943	NA	NA	NA	NA	NA
NA	NA	NA	NA	NA	NA
NA	NA	1.040616371	NA	NA	1.174562797
NA	NA	NA	NA	NA	403.5157649
NA	NA	NA	NA	NA	NA
NA	NA	NA	NA	NA	NA
NA	NA	NA	NA	NA	NA
NA	NA	NA	NA	NA	NA
NA	NA	0.922237257	NA	NA	NA
NA	NA	NA	NA	NA	NA
1.021582252	NA	1.01933389	NA	NA	NA
NA	NA	NA	NA	NA	NA
NA	NA	NA	NA	NA	NA
NA	NA	NA	NA	NA	NA
NA	1.003522153	NA	1.006911935	NA	NA
1.039068389	NA	NA	NA	NA	NA
NA	NA	NA	NA	NA	163481483
NA	NA	1.024080518	NA	NA	NA
0.957011836	1.051701331	NA	NA	0.918870166	NA
NA	NA	NA	NA	NA	NA
1.022832587	NA	1.055643189	NA	NA	NA
NA	NA	1.042906796	NA	NA	NA
NA	NA	NA	NA	NA	NA
NA	NA	0.982611171	NA	NA	NA
NA	NA	NA	NA	NA	NA
NA	NA	NA	NA	NA	NA
NA	NA	NA	NA	NA	NA
NA	NA	NA	0.467142828	NA	NA

PRAD	READ	SARC	SKCM	STAD	TGCT
NA	NA	NA	NA	NA	NA
NA	0.50888624	NA	NA	NA	NA
NA	NA	NA	NA	2.762495328	NA
NA	NA	NA	NA	1.057933739	NA
NA	NA	2.103949712	NA	NA	NA
NA	NA	NA	NA	NA	NA
1.267104961	NA	NA	NA	NA	NA
NA	NA	NA	NA	NA	NA
1.627895359	69.74516632	NA	NA	NA	NA
1.016918133	NA	1.021766176	NA	NA	NA
NA	NA	NA	NA	NA	NA
NA	NA	NA	NA	NA	NA
NA	NA	NA	NA	NA	NA
NA	NA	NA	NA	NA	NA
NA	NA	NA	NA	NA	NA
NA	NA	1.012296945	NA	NA	NA
NA	NA	NA	NA	NA	NA
NA	NA	NA	1.048221336	NA	NA
1.595397854	NA	NA	NA	NA	NA
NA	NA	NA	NA	NA	NA
NA	NA	1.235888391	NA	NA	NA
NA	NA	1.181292449	NA	NA	NA
NA	NA	NA	NA	1.045048065	NA
NA	NA	NA	NA	NA	NA
NA	NA	NA	NA	NA	NA
NA	NA	NA	NA	NA	NA
NA	NA	NA	NA	NA	NA
NA	NA	NA	NA	NA	NA
NA	NA	NA	0.998368453	NA	NA
NA	NA	1.281437988	NA	NA	NA
NA	NA	NA	NA	NA	NA
NA	NA	NA	NA	NA	NA
NA	NA	NA	NA	NA	NA
NA	NA	NA	NA	NA	NA
NA	0.977574362	NA	NA	NA	NA
NA	NA	NA	NA	NA	NA
NA	0.800240527	NA	NA	NA	NA
NA	NA	1.096342519	NA	2.143907961	NA
NA	NA	NA	NA	NA	NA
NA	NA	NA	NA	NA	NA
NA	NA	NA	0.97845145	NA	NA
NA	NA	NA	NA	1.157649543	NA
NA	2.049931419	NA	NA	NA	NA

NA	NA	NA	0.937911737	NA	NA
NA	1.043886822	NA	NA	NA	NA
NA	NA	1.373435714	NA	NA	NA
NA	NA	NA	NA	NA	NA
NA	NA	NA	NA	NA	NA
NA	NA	NA	0.988792243	NA	1.132756866
NA	NA	NA	NA	NA	NA
NA	NA	NA	NA	NA	NA
NA	NA	NA	NA	NA	NA
NA	NA	NA	NA	NA	NA
NA	NA	1.187366836	NA	NA	NA
NA	NA	1.164781582	0.957201017	NA	NA
NA	NA	1.001064667	NA	NA	NA
NA	NA	NA	NA	0.98320524	NA
NA	NA	NA	NA	NA	NA
NA	NA	NA	NA	NA	NA
NA	NA	NA	NA	1.038586303	NA
NA	3.647119149	NA	NA	NA	NA
NA	NA	1.350306352	NA	NA	NA
NA	NA	NA	NA	NA	NA
NA	NA	NA	NA	1.074303364	NA
NA	NA	NA	NA	NA	NA
NA	NA	NA	NA	NA	NA
1.386034402	NA	NA	NA	NA	NA
NA	NA	NA	1.017312051	NA	NA
NA	NA	NA	NA	NA	NA
NA	NA	NA	NA	NA	NA
NA	NA	NA	0.761784138	NA	NA
NA	NA	NA	NA	NA	NA
NA	NA	NA	NA	NA	NA
NA	NA	NA	NA	NA	NA
NA	1.331039233	NA	1.012359231	NA	NA
NA	NA	NA	NA	NA	NA
NA	0.740031959	NA	NA	NA	NA
NA	NA	NA	NA	NA	NA
NA	1.28464072	NA	NA	NA	NA
NA	NA	NA	NA	NA	NA
NA	1.020782403	NA	NA	0.986520398	NA
NA	NA	1.09723484	1.048558348	NA	NA
NA	NA	NA	NA	NA	NA
NA	NA	NA	NA	NA	NA
NA	NA	NA	0.83410422	NA	1.780183213

THCA	THYM	UCEC	UVM
NA	NA	NA	NA
2.080892587	NA	NA	NA
NA	NA	NA	91889.7625
1.049578473	NA	NA	NA
NA	NA	NA	NA
NA	NA	NA	NA
NA	NA	NA	NA
NA	NA	NA	NA
NA	NA	NA	NA
1.064225211	NA	NA	NA
NA	0.36165627	1.269593596	NA
NA	NA	NA	1.195691857
NA	NA	NA	NA
NA	1.0803979	NA	1.121470733
1.022916759	NA	NA	NA
NA	NA	1.01928599	NA
NA	NA	NA	NA
NA	NA	NA	NA
1.273346529	NA	NA	NA
NA	NA	NA	NA
NA	5.37666871	NA	NA
NA	NA	NA	NA
NA	NA	NA	NA
NA	NA	NA	NA
NA	NA	NA	NA
NA	NA	NA	1.837032701
NA	NA	NA	NA
NA	NA	NA	36.68048312
1.040698225	NA	NA	1.220271066
NA	NA	NA	NA
NA	NA	NA	NA
NA	NA	NA	NA
NA	1565.172624	NA	NA
1.04309008	1.210730464	NA	NA
NA	0.97237509	NA	NA
NA	NA	NA	NA
NA	NA	NA	NA
NA	NA	2.101506193	NA
NA	0.173537579	NA	NA
NA	NA	NA	NA
NA	NA	NA	NA
NA	NA	NA	NA
NA	NA	NA	NA

NA	NA	NA	NA
NA	NA	NA	NA
NA	NA	0.20750912	1.971523716
NA	NA	NA	NA
13.54852907	0.214517721	0.939306303	NA
NA	NA	NA	NA
1.006874674	NA	NA	NA
NA	NA	NA	NA
NA	NA	NA	NA
NA	0.355847735	NA	NA
NA	NA	NA	NA
NA	NA	NA	NA
NA	NA	NA	NA
NA	NA	NA	1.310452697
NA	NA	NA	NA
NA	NA	NA	NA
NA	NA	NA	NA
NA	1.443422281	1.201520826	NA
NA	NA	NA	NA
NA	NA	NA	NA
NA	NA	NA	NA
NA	0.651905848	NA	NA
NA	NA	NA	NA
NA	NA	NA	NA
NA	NA	NA	NA
1.006171789	NA	NA	NA
NA	1.338004365	NA	0.754080484
NA	1.485230435	NA	NA
1.017780927	NA	NA	NA
NA	NA	NA	NA
NA	33495.71027	NA	NA
1.233118977	NA	NA	NA
NA	NA	NA	NA
NA	NA	NA	NA
1.095102221	NA	NA	NA
0.977407995	NA	NA	1.924014922
NA	NA	NA	NA
NA	NA	NA	NA
NA	NA	NA	NA
NA	NA	NA	NA
1.037121351	NA	NA	NA
NA	1.515011942	NA	NA
